# ER stress response plays an important role in aggregation of α-synuclein

**DOI:** 10.1186/1750-1326-5-56

**Published:** 2010-12-13

**Authors:** Peizhou Jiang, Ming Gan, Abdul Shukkur Ebrahim, Wen-Lang Lin, Heather L Melrose, Shu-Hui C Yen

**Affiliations:** 1 Department of Neuroscience, Mayo Clinic, 4500 San Pablo Road, Jacksonville, Florida 32224, USA

## Abstract

**Background:**

Accumulation of filamentous α-synuclein as Lewy bodies is a hallmark of Parkinson's disease. To identify the mechanisms involved in α-synuclein assembly and determine whether the assemblies are cytotoxic, we developed a cell model (3D5) that inducibly expresses wild-type human α-synuclein and forms inclusions that reproduce many morphological and biochemical characteristics of Lewy bodies. In the present study, we evaluated the effects of several histone deacetylase inhibitors on α-synuclein aggregation in 3D5 cells and primary neuronal cultures. These drugs have been demonstrated to protect cells transiently overexpressing α-synuclein from its toxicity.

**Results:**

Contrary to transient transfectants, the drug treatment did not benefit 3D5 cells and primary cultures. The treated were less viable and contained more α-synuclein oligomers, active caspases 3 and 9, as well as ER stress markers than non-treated counterparts. The drug-treated, induced-3D5 cells, or primary cultures from transgenic mice overexpressing (<2 fold) α-synuclein, displayed more α-synuclein oligomers and ER stress markers than non-induced or non-transgenic counterparts. Similar effects were demonstrated in cultures treated with tunicamycin, an ER stressor. These effects were blocked by co-treatment with salubrinal, an ER stress inhibitor. In comparison, co-treatment with a pan caspase inhibitor protected cells from demise but did not reduce α-synuclein oligomer accumulation.

**Conclusions:**

Our results indicate that an increase of wild-type α-synuclein can elicit ER stress response and sensitize cells to further insults. Most importantly, an increase of ER stress response can promote the aggregation of wild type α-synuclein.

## Background

Parkinson's disease (PD) and related disorders known as synucleinopathies are characterized by abnormal accumulation of α-synuclein (α-Syn). Such filamentous aggregates in neuronal perikarya and processes are referred to as Lewy bodies and Lewy neurites, respectively [1,2]. Most synucleinopathies are sporadic, but some are linked to missense mutation or multiplications of the gene encoding α-Syn [3-8].

In order to understand how and why α-Syn accumulation affects neuronal survival, several experimental models have been generated. In some cell-based studies overexpression of α-Syn did not affect cell survival but sensitized the cultures (mutant α-Syn in particular) to insults [9,10]. In others, the results were different. Infection of primary mesencephalic neuronal cultures with lentiviral constructs containing A53T mutant α-Syn led to reduced cell viability [11]. Transient co-expression of mutant α-Syn and synphilin-1 in H4 cells was reported to cause formation of cytoplasmic α-Syn inclusions and cytotoxicity. These changes were alleviated by treatment with Agk2, a histone deacetylase (HDAC) inhibitor [11]. Transient expression of wild-type α-Syn in the nuclei of SH-SY5Y cells, but not cytoplasm, was reported to cause α-Syn aggregation and cytoxicity that can be prevented by a 48-hour (h) treatment with 10 mM sodium butyrate (SB) [12]. It remains unknown whether benefits comparable to those observed in transient transfectants are attainable in stable transfectants overexpressing α-Syn and whether comparable outcomes can be achieved with exposure to other HDAC inhibitors, since there are multiple classes of HDAC. Some of these issues were examined in the present studies using cell cultures, referred to as 3D5, of human neuroblastoma BE2-M17D cells overexpressing wild-type human α-Syn [13]. The overexpression of human α-Syn in 3D5 cultures is regulated by the tetracycline-off (TetOff) inducible mechanism. These cells develop neuritic processes and express neuronal markers in response to all-trans-retinoic acid (RA) treatment [14]. 3D5 cells that have been treated with RA for 10 days (ds) followed by 28 ds of induced α-Syn expression are capable of forming α-Syn inclusions that reproduce many morphological and biochemical characteristics of Lewy bodies [14]. Similar α-Syn inclusions were not readily detected in 3D5 cultures after 14 ds of induced α-Syn expression, although the cultures were biochemically demonstrated to contain α-Syn oligomers.

It has been well documented that acetylation and deacetylation of histone proteins play important roles in the epigenetic regulation of transcription and other cellular functions [15,16]. Deacetylation of histone proteins leads to the silencing of gene expression. At least four major classes of HDACs have been identified in mammalian cells [17-21] and several HDAC inhibitors have been developed. Some HDAC inhibitors are more specific to certain subclasses of HDAC than others [17,18]. For example, fatty acid derivatives such as sodium butyrate (SB) and valproic acid (VPA) are capable of inhibiting class I (HDAC8 in particular) and class II HDAC [22]. In comparison, Agk2 is more specific to sirtuin 2, a member of class III HDAC [11].

In this study we used RA-differentiated 3D5 cultures, with or without induced α-Syn expression, and evaluated their responses to SB, VPA or Agk2 exposure. We began with cultures that have 10 ds of induced α-Syn expression. The goal was to establish background information on the drug dosage and duration of treatment and use the information in a subsequent cell-based study with an extended duration (e.g 28 ds) of α-Syn overexpression. We unexpectedly found that SB or other HDAC inhibitors treatment of 3D5 cells with induced α-Syn expression caused an increase of α-Syn aggregate accumulation in a time- and dose-dependent manner. Such changes were not detected in the SB-treated, non-induced cultures or in 3D5 cells treated with sodium acetate (SA). More important, 3D5 cultures with α-Syn induction were less viable than those without the induction when exposed to HDAC inhibitors. Similar results were obtained using primary neuronal cultures generated from α-Syn transgenic (TG) mice and their non-transgenic (NT) littermates. The TG mice express wild-type human α-Syn at levels much lower than that displayed by the TetOff induced 3D5 cultures. These findings prompted us to examine whether α-Syn aggregation is closely associated with apoptosis or upstream pathways.

## Results

### SB exposure causes accumulation of α-Syn oligomers and activation of caspase 3 in a dose- and time-dependent manner

**Figure 1:**
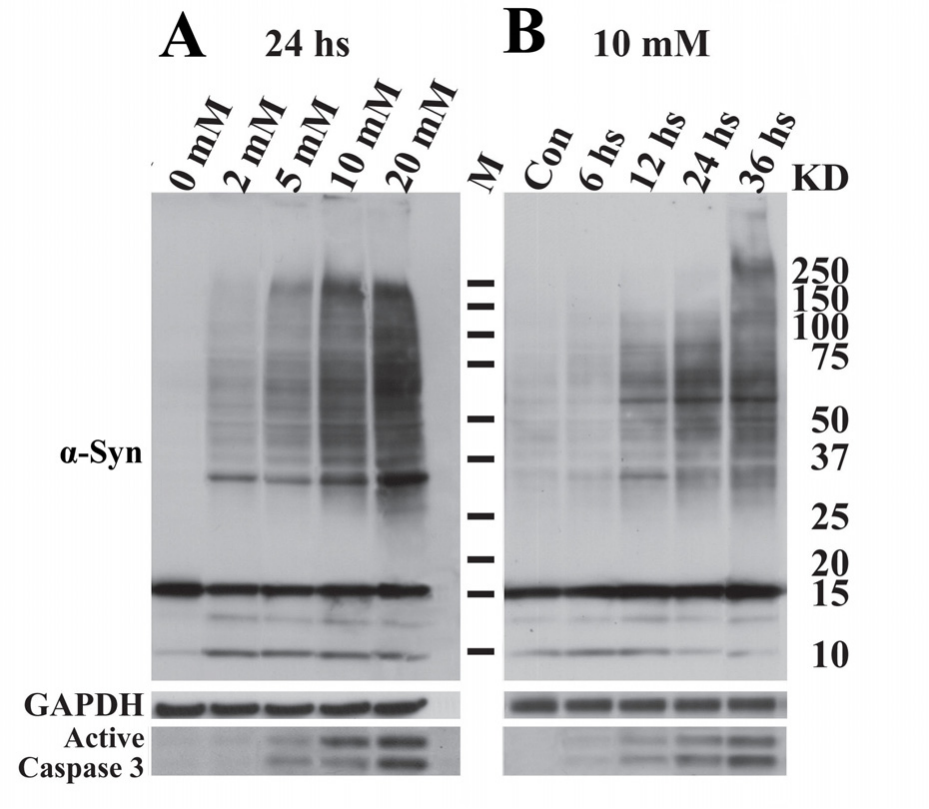
**Effects of sodium butyrate (SB) exposure on α-Syn oligomerization and caspase 3 activation**. 3D5 cells with 10 days (ds) of RA-elicited differentiation and TetOff-induced α-Syn expression were exposed to **(A) **0, 2, 5, 10 or 20 mM SB for 24 hours (hs) and **(B) **10 mM SB for 6, 12, 24 or 36 hs. Cultures without SB treatment for 36 hs were used as controls (Con) for **(B)**. Proteins from the SB-treated and non-treated cultures were resolved by SDS-PAGE and probed with antibodies to α-Syn, GAPDH and active forms of caspase 3. The SB-treated cultures contained more α-Syn oligomers (34 to 230 kDa in size) and cleaved caspase 3. The effect of SB on α-Syn oligomerization and apoptosis is dose- and time-dependent. Molecular weight markers shown in current and subsequent figures were for determining the size of α-Syn products.

We exposed 3D5 cultures to between 2 and 20 mM SB for 24 hrs and determined the effects of these exposures on α-Syn oligomer accumulation. This drug is commonly used at a millimolar range and has been shown to inhibit histone deacetylation in cell cultures at 2 mM or higher concentrations [23]. Prior to the drug treatment, 3D5 cultures were differentiated with RA and induced to express α-Syn for 10 ds. Compared to non-treated cultures, the drug treatment caused accumulation of different sized (≥34 kDa) α-Syn oligomers in a dose-dependent manner as demonstrated by Western blotting using α-Syn antibodies Synuclein 1 (Figure 1A) and Ab 98 (data not shown), which recognize different epitopes. This increase in α-Syn oligomer levels was accompanied by increased amounts of active caspase 3 (Figure 1A). We also analyzed cell lysates from cultures treated with 10 mM SB for different durations. We found that the levels of α-Syn oligomers and active caspase 3 increased in a time-dependent manner (Figure 1B). Similar results were demonstrated in 3D5 cultures exposed to VPA or Agk2 (Additional file 1). In contrast, exposure of 3D5 cells to sodium acetate (SA, also a derivative of short-chain fatty acid) did not increase α-Syn oligomer accumulation (Additional file 2).

### Cultures with SB exposure accumulate α-Syn oligomers with different solubility

**Figure 2:**
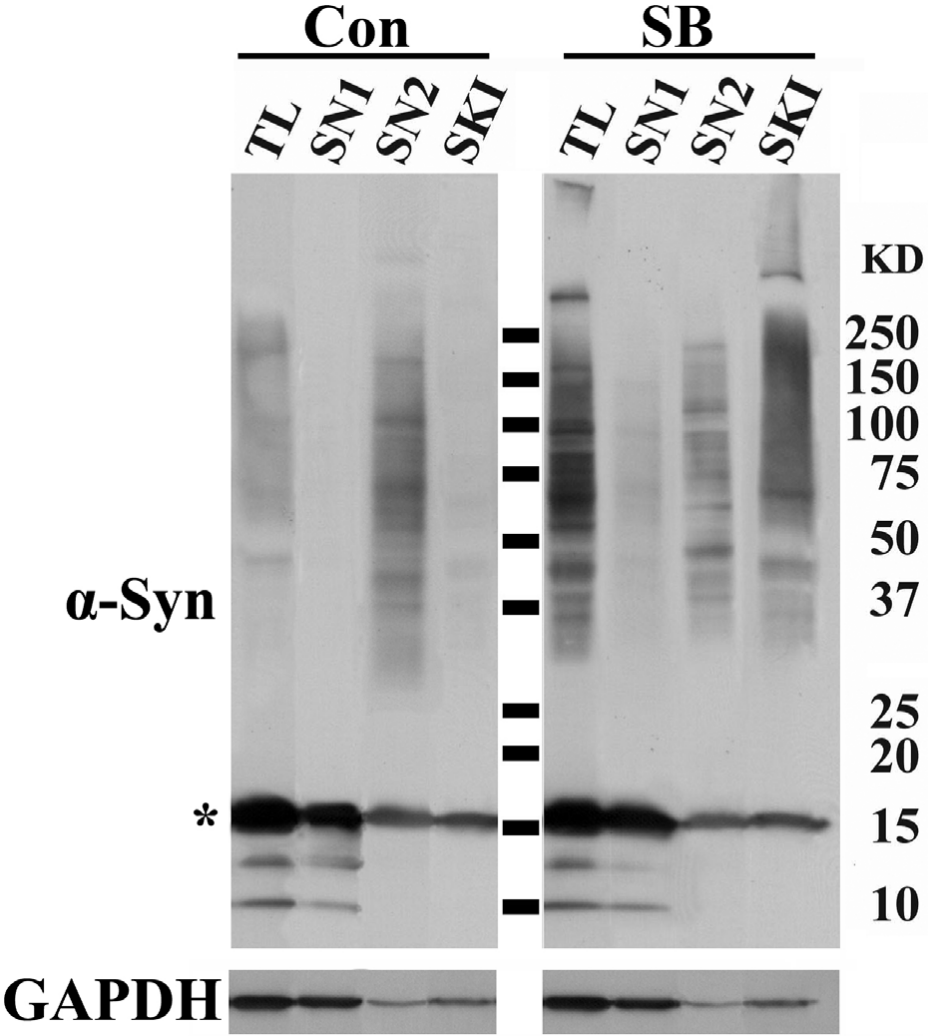
**SB-treated cultures accumulate α-Syn of different solubility and sizes**. 3D5 cells were treated with 10 mM SB for 36 hs after 10 ds of differentiation and induced α-Syn expression. Cultures without the drug treatment were used as controls (Con). Total cell lysates (TL) were fractionated to obtain buffer-soluble (SN1), buffer-insoluble and salt plus sarkosyl-soluble (SN2) as well as sarkosyl-insoluble (SKI) fractions. These samples were evaluated by immunoblotting using TL:SN1:SN2:SKI at 1:1:3.75:9.37 loading and antibodies to α-Syn. GAPDH was used as loading control. Asterisk marks monomeric α-Syn.

To characterize α-Syn oligomers further, we fractionated total lysates (TL) from non-treated (control) and SB-treated 3D5 cultures to generate buffer-soluble (SN1), salt plus sarkosyl-extractable (SN2) and sarkosyl-insoluble (SKI) samples and then analyzed using Western blotting (Figure 2). All fractions from SB-treated cells contained α-Syn oligomers (Figure 2), although the SKI fraction displayed more large-sized oligomers, some of which were retained at the top of separation gel. After correction for differences in sample loading, the amount of α-Syn oligomers recovered in SKI fraction was about the same as that recovered in the SN2 or SN1 fraction. In comparison, samples from non-treated cells contained much less sarkosyl-insoluble α-Syn oligomers.

### Morphological assessment of cultures and SKI samples

#### Immunocytochemistry

**Figure 3:**
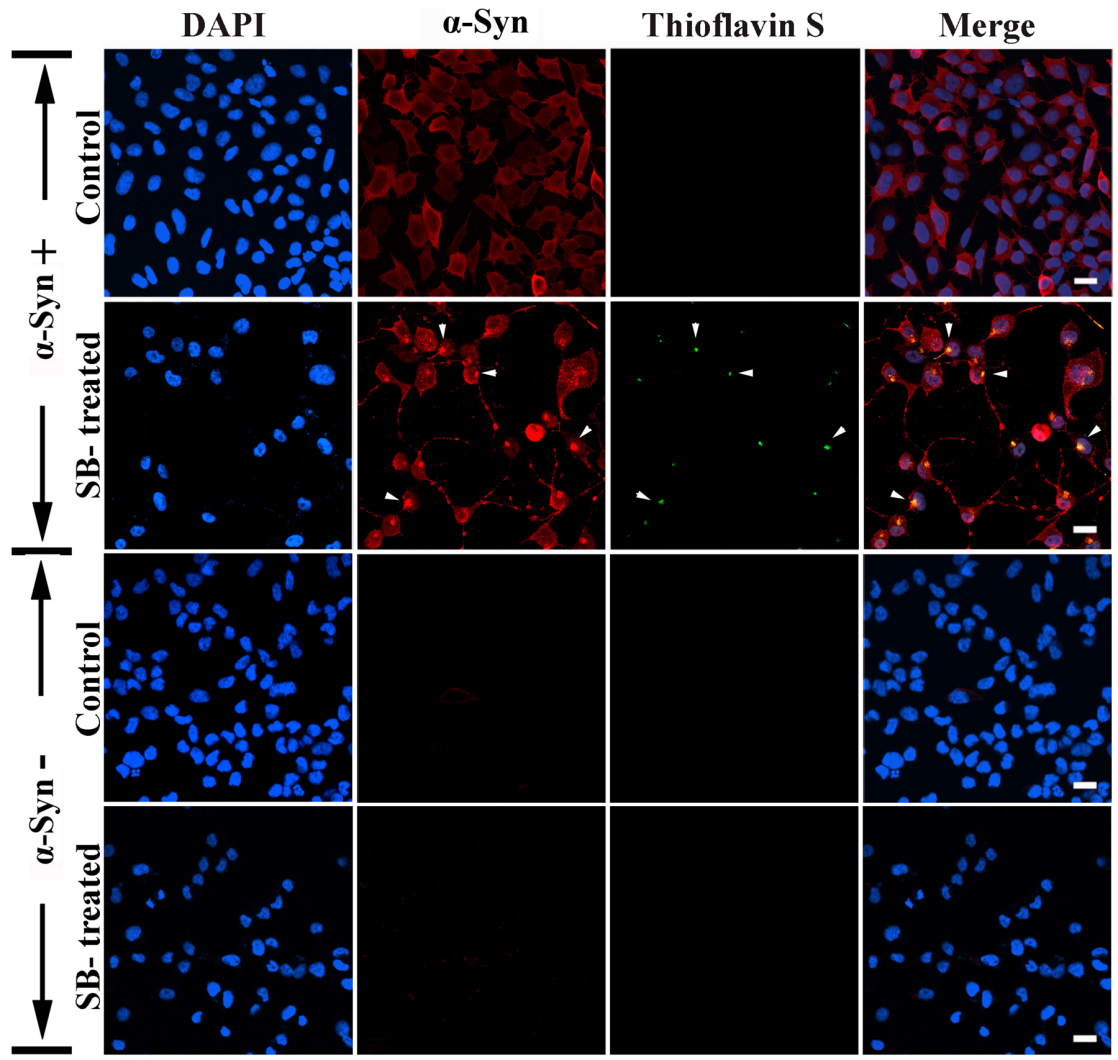
**Accumulation of α-Syn and thioflavin-positive inclusions in SB-treated cultures**. Retinoic acid-differentiated 3D5 cells with induced α-Syn expression (α-Syn+) or without (α-Syn-) were treated with SB (10 mM) or without (control) for 36 hs, followed by α-Syn immunofluorescence labeling and Thioflavin S staining. Alpha-Syn and Thioflavin S positive inclusions were detected only in the SB-treated cultures (marked by arrowheads). Nuclei were marked with DAPI stain. About 50% of cells with α-Syn expression and SB treatment contained aggregated α-Syn, and at the least 40% of such aggregates were Thioflavin S positive. Scale bar: 20 μm

To determine whether SB exposure during α-Syn induction (α-Syn+) promotes accumulation of α-Syn aggregates and subsequent fibrillogenesis, 3D5 cells were subject to immunocytochemistry with α-Syn antibodies and Thioflavin S staining. Many SB-treated cells displayed both α-Syn and Thioflavin positive inclusions (Figure 3, SB-treated, marked by arrowheads), however, such a labeling pattern was neither demonstrated in α-Syn+ cultures without SB exposure nor in α-Syn-cultures with SB treatment (Figure 3, Control).

#### Electron microscopy

**Figure 4:**
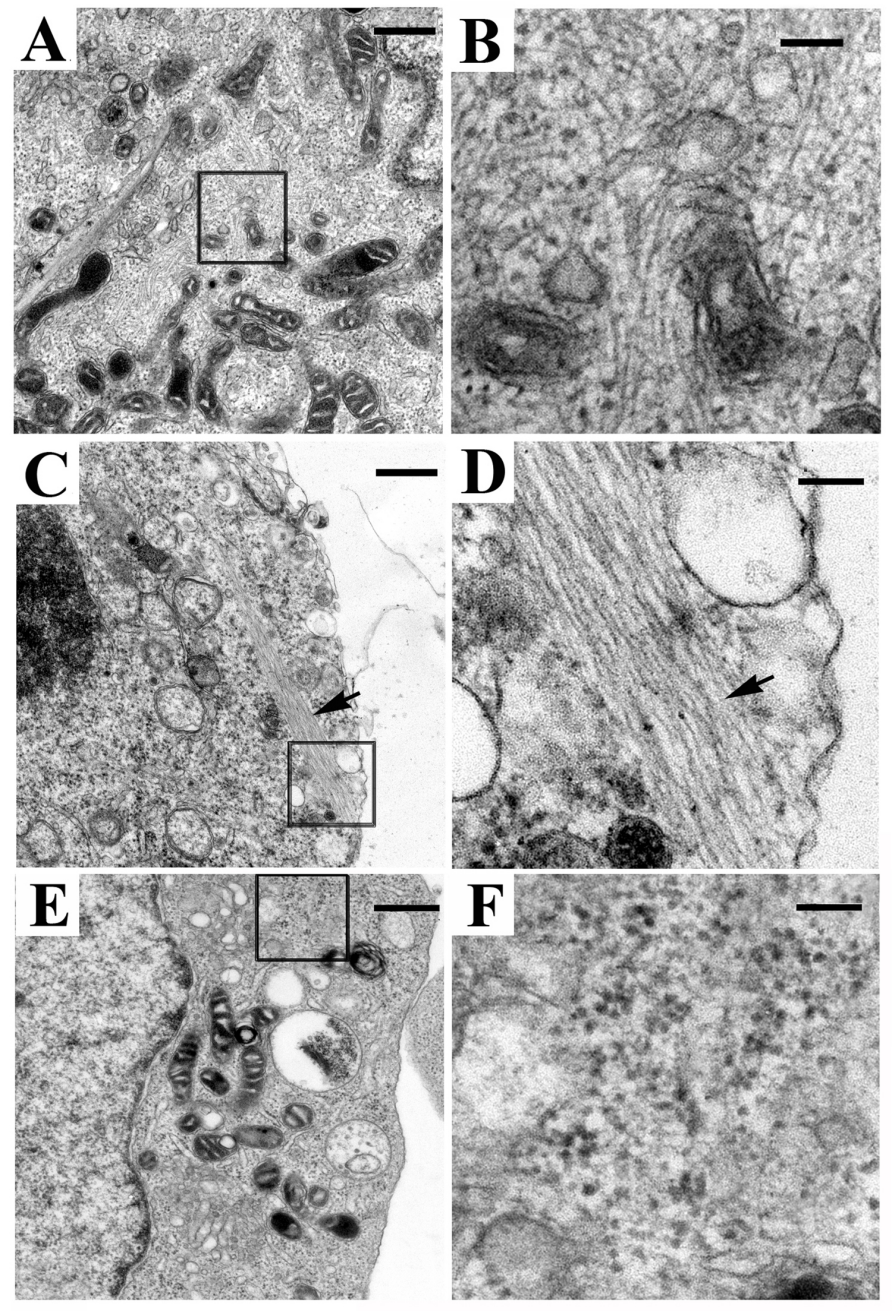
**Electron micrographs of cultures with and without drug treatment**. **(A-D) **Exposure to SB (10 mM) for 36 hs resulted in accumulation of filamentous aggregates in differentiated 3D5 cells with induced α-Syn expression. **(E-F) **Without the SB treatment very few filamentous elements were detected in differentiated 3D5 cells with induced α-Syn expression. **(B)**, **(D) **and **(F) **are higher magnification photographs of the framed area shown in **(A)**, **(C) **and **(E)**, respectively. The filaments have a diameter of either **(B) **8-10 nm or **(D) **around 5 nm. Scale bar: 0.5 μm (A, C and E); 100 nm (B, D and F).

The SB-treated α-Syn+ cells contained two groups of filamentous structures, which appeared in clusters or bundles (Figures. 4A-D). These filaments had a diameter of either 8-10 nm or about 5 nm (Figures. 4B &4D), and are far less abundant in α-Syn+ cultures without the SB treatment (Figures. 4E &4F).

#### Immunoelectron microscopy

**Figure 5:**
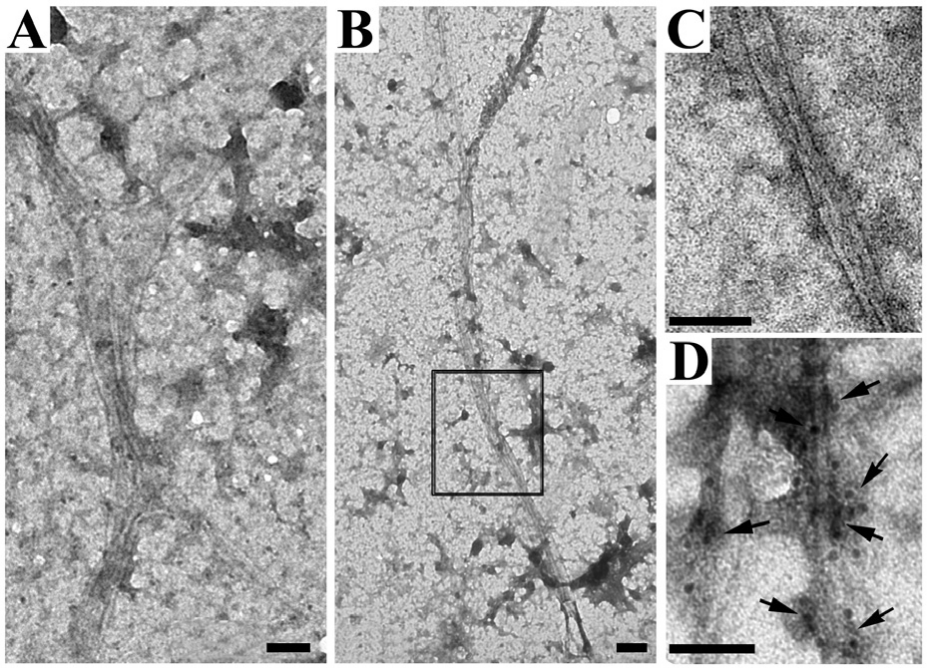
**Ultrastructural evaluation of sarkosyl-insoluble preparations from cell cultures**. **(A-C) **Sarkosyl-insoluble samples from SB-treated 3D5 cells depicted in Fig. 2 were adsorbed on EM grids and negatively stained with 5% uranyl acetate. They contain bundles of filamentous assemblies of diameter about 8-10 nm. The framed area in **(B) **is shown in **(C) **at higher magnification. **(D) **The filaments were decorated with 5-nm gold particles (arrows) by immunogold labeling using an anti-α-Syn antibody, thus verifying that they were assembled from α-Syn. Similar filamentous structures were not detected in cultures without the SB treatment (data not shown). Scale bar: 100 nm

To determine whether SB exposure resulted in assembly of filamentous α-Syn, we probed SKI samples prepared from cultures with either α-Syn antibodies or without, followed by secondary antibodies with immunogold labeling and viewed at the ultrastructural level. Filamentous elements of diameter 8-10 nm were detected in samples from the SB-treated samples only, and they often appear as small bundles (Figure 5A-C) positive for α-Syn (Figure 5D, arrows marked immunogold labeled filaments). In contrast, 5 nm filaments were not detected in these samples by electron or immunoelectron microscopy.

### SB exposure does not affect the level of α-Syn in 3D5 cells with induced α-Syn expression

**Figure 6:**
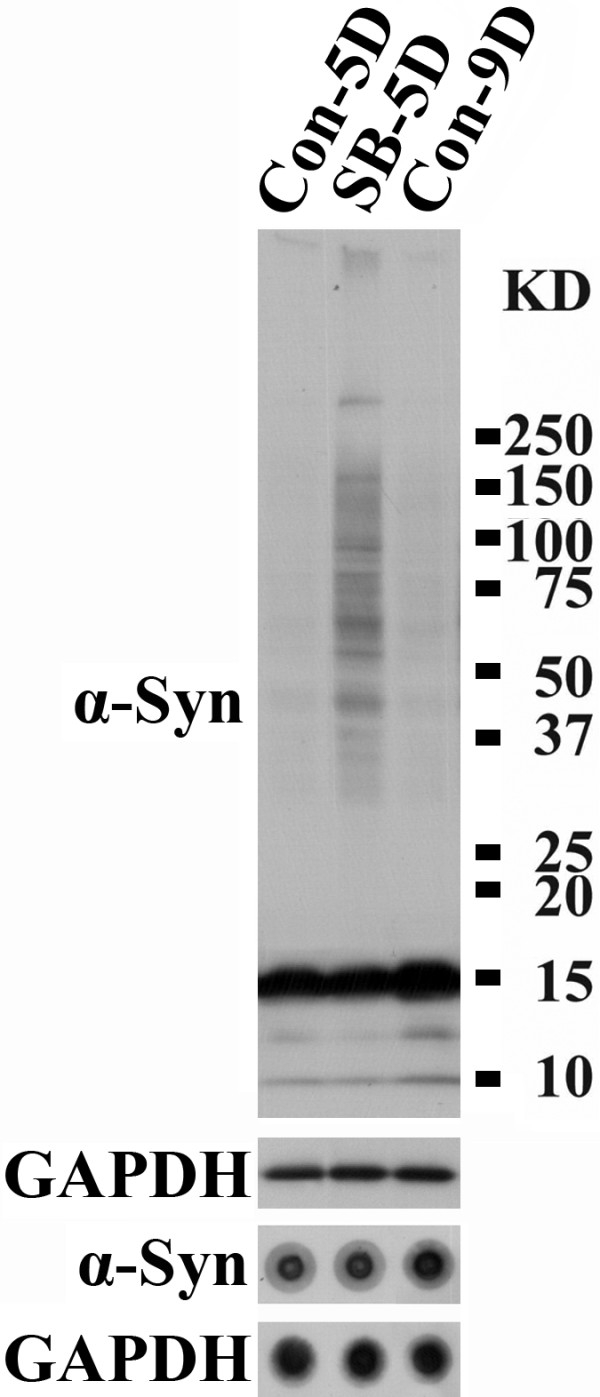
**The effect of SB exposure on α-Syn levels in TetOff-induced cultures is minimal**. One day after the seeding of 3D5 cells RA was added to the cultures to elicit differentiation for 10 ds. Two subsets of these cultures were induced after 5 ds of differentiation to express α-Syn for 5 ds. Another set was induced after 1 d of differentiation to express α-Syn for 9 days. One set of the 5 d-induced cultures was exposed to SB for 24 hs before cell harvesting (labeled as SB-5D). Cultures with 5 and 9 ds of induced α-Syn expression and without SB exposure are marked as Con-5D and Con-9D, respectively. Cell lysates containing the same amount of proteins from different samples derived from 3 independent experiments were used for dot blotting with antibodies to α-Syn and GAPDH, or Western blotting. Quantitative analyses were carried out on dot blots to determine the ratio of α-Syn to GAPDH. When the ratio derived from Con-5D dot blots was set as 1, the relative amount of α-Syn to GAPDH is 0.97 ± 0.09 (average ± standard error of mean) and 1.58 ± 0.06 for SB-5D and Con-9D preparations, respectively. Based on Student's t-test, there are no significant differences (P > 0.8) between Con-5D and SB-5D. In comparison, there are significant differences (P < 0.001) between Con-9D and SB-5D or Con-5D. Since α-Syn oligomers were readily detected only in SB-5D cultures, their production/accumulation is not caused by an increase in the expression of α-Syn alone.

It has been reported that a major cellular effect of SB is modulation of gene expression [15,16]. Therefore, the accumulation of α-Syn oligomers in SB-treated 3D5 cultures may be due to an increase of α-Syn expression. This possibility was tested by comparing the amount of total, monomeric and oligomeric α-Syn in the SB-treated and non-treated cultures, which had been subjected to 10 ds of differentiation plus 9 ds of induced α-Syn expression or 10 ds of differentiation plus 5ds of induction (started on the 5^th ^day of differentiation). By dot blotting (Figure 6), we detected very few differences in α-Syn expression between the 5 d-induced cultures with and without SB treatment, but found higher levels of α-Syn in the 9 d-induced cultures. Based on 3 independent experiments, in which the ratio of α-Syn to GAPDH was set to 1 for 5d-induced samples, the ratio is 0.97 ± 0.09 (average ± stand error of mean) and 1.58 ± 0.06, for the 5d-induced with SB treatment and the 9-d induced cultures, respectively. Statistical analysis demonstrated a significant difference (P < 0.001) between the 5d- and 9d-induced samples but no difference (P > 0.8) between the 5d-induced and its SB-treated counterpart. More α-Syn oligomers were observed by Western blot in the 5 d-induced, SB-treated cultures than the 9 d-induced, non-treated. The results indicate that a 24-h exposure of 3D5 cells to SB does not affect α-Syn levels, suggesting that other factors, besides α-Syn expression, may be involved in the accumulation of α-Syn oligomers in cultures with SB treatment.

To demonstrate further that SB treatment did not affect oligomeric α-Syn accumulation via an increase of α-Syn synthesis, we analyzed 3D5 cells that have been exposed to SB, SB plus Tet (2 μg/ml, for blocking of α-Syn induction), SB plus cycloheximide (CHX, 20 μM, for inhibition of protein synthesis), CHX or Tet for 36 hs by Western blotting. The analysis showed the co-treated cultures contained only slightly fewer α-Syn oligomers than those treated with SB, but much more than those treated with CHX or Tet (Additional file 3). The results support the possibility that oligomer accumulation in SB-treated cells is unlikely caused by an increase of α-Syn expression.

### Caspase inhibitor treatment has no affect on α-Syn oligomer accumulation

**Figure 7:**
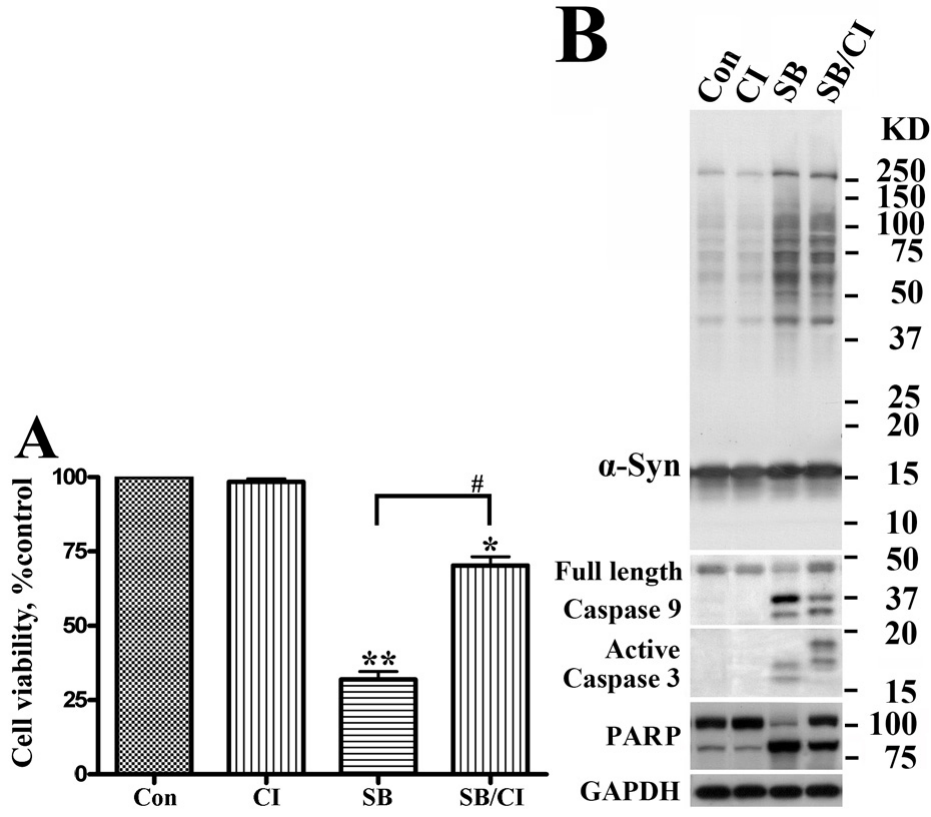
**Pan-capase inhibitor treatment reduces the effect of SB on apoptosis but not α-Syn oligomer accumulation**. 3D5 cells with 10 ds of differentiation and induced α-Syn expression were treated with 10 mM SB in the presence or absence of 50 μM z-VAD (OMe)-fmk, a pan caspase inhibitor (CI), for 24 hs. Cultures without any drug treatment or treated with CI only were included as controls. **(A) **Calcein AM cell viability assay. The SB-treated cultures displayed a significant decrease of cell viability when compared to untreated counterparts (**P < 0.01, n = 3). The extent of cell death is significantly reduced (#P < 0.05, n = 3) in cultures treated with SB plus CI. **(B) **Western blotting of cell lysates. Antibodies to α-Syn, caspase 9, active Caspase 3, PARP and GAPDH were used. The SB and CI co-treated or the SB-treated cultures displayed similar levels and profiles of α-Syn oligomers and contained more α-Syn oligomers than non-treated (Con) or CI-treated control. In contrast, the SB and CI co-treated cultures displayed more full-length and less cleaved caspases 3 and 9 or PARP than the SB-treated. Molecular weight markers were included as references.

To determine the effect of SB treatment on cell viability, we treated subsets of differentiated and induced 3D5 cultures with SB or SB plus a pan caspase inhibitor (CI). Cultures without any drug treatment or treated only with CI were included as controls. The SB exposure caused a significant decrease in cell viability (Figure 7A). Such effect was significantly attenuated in cultures co-treated with CI, indicating a role for apoptosis in SB cytotoxicity. Treatment of cultures with CI alone did not affect their viability.

We next determined whether the SB-treated cultures differed from non-treated in terms of apoptosis and whether apoptosis *per se *is responsible for accumulation of α-Syn oligomers (Figure 7B). The SB-treated cultures were found to contain far more active forms of capases 3, caspase 9 and cleaved PARP than the non-treated, indicating the treated underwent apoptosis. In cells co-treated with SB and CI, the amount of both active capases 3 and 9 was reduced, whereas the level of α-Syn oligomers was not, indicating α-Syn aggregation is likely an upstream event of caspase activation.

### Exposure of α-Syn overexpressing 3D5 cultures to SB or other HDAC inhibitors induces ER stress response leading to accumulation of α-Syn oligomers and apoptosis

**Figure 8:**
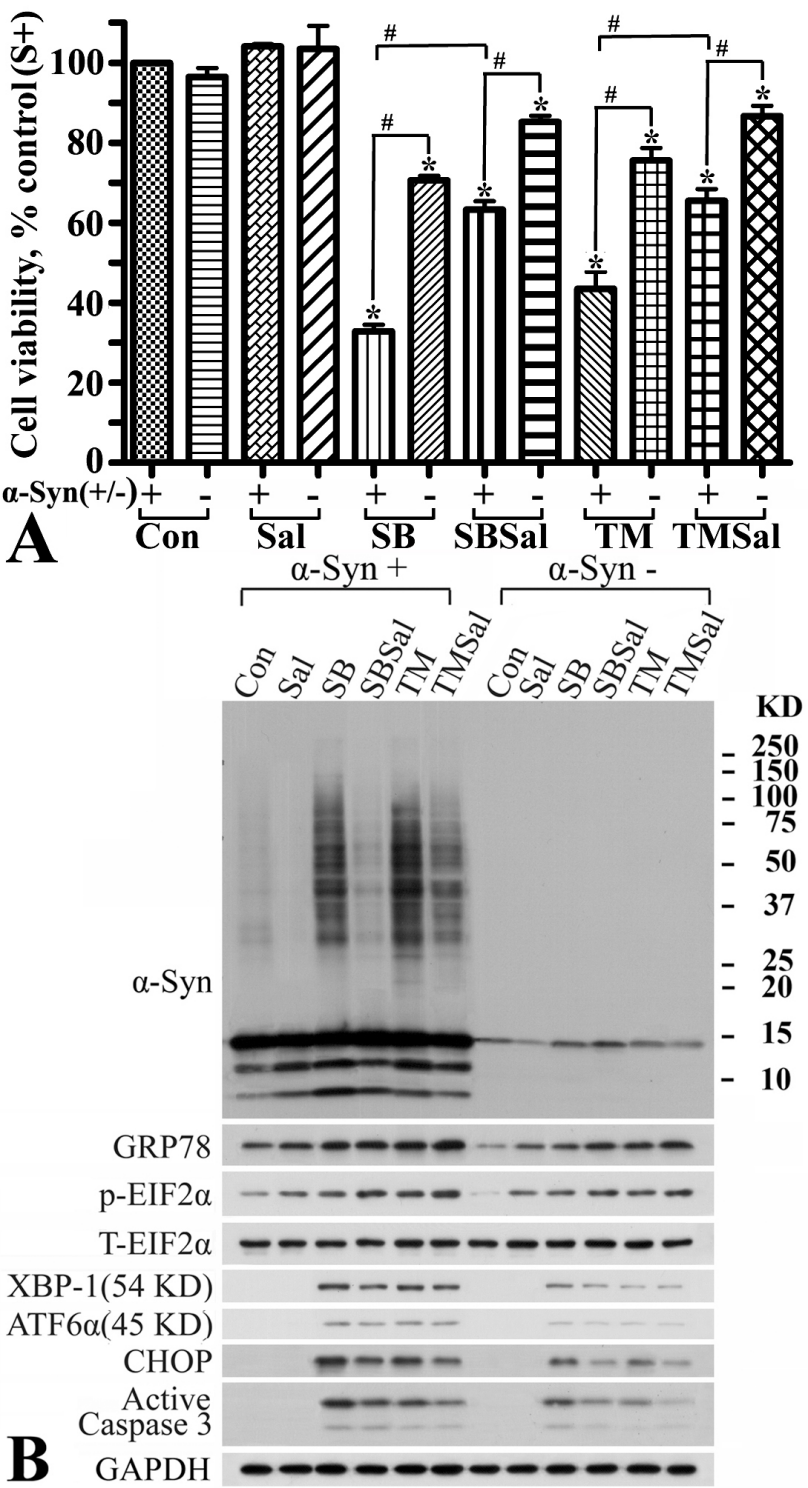
**Association of α-Syn oligomer accumulation with ER stress response**. Differentiated 3D5 cells with (α-Syn +) or without (α-Syn -) induced α-Syn expression were treated with 10 mM SB (10 mM), 40 μM salubrinal (Sal, an inhibitor of EIF2α dephosphorylation), 10 μg/ml tunicamycin (TM, an ER stress inducer), SB plus Sal (SBSal) or TM plus Sal (TMSal) for 36 hs. Cells without any drug treatment served as controls. **(A) **Cell viability. Significant differences were detected between drug treated and non-treated cultures [*P < 0.05 (e.g. SB/α-Syn + vs. Con/α-Syn +, SB/α-Syn - vs. Con/α-Syn -)], between drug treated α-Syn + and α-Syn- cultures [#P < 0.05 (e.g. SB/α-Syn + vs. SB/α-Syn -)] or between SB (or TM) and SB (or TM) plus Sal treated cultures [#P < 0.05 (e.g. SB/α-Syn + vs. SBSal/α-Syn +, TM/α-Syn + vs. TMSal/α-Syn +)]. **(B) **Western blotting. Samples from cultures with different treatments were probed with antibodies to α-Syn, six markers of ER stress, active caspase 3 and GAPDH. Exposure of α-Syn+ cultures to SB or TM promoted accumulation of α-Syn oligomers and expression of most ER stress markers. The presence of Sal, however, inhibited this effect (i.e. SB versus SBSal, or TM versus TMSal). Similar drug exposure affected the ER stress response less in α-Syn- cultures than in α-Syn+ cultures.

Exposure to HDAC inhibitors such as SB has been reported to cause not only apoptosis, but also lipid peroxidation [24], oxidative stress [24-27], mitochondrial dysfunction [28,29] and ER stress [30-32]. Since accumulation of α-Syn oligomers in 3D5 cells was not blocked by treatment with pan caspase inhibitor, we decided to test whether it can be reduced by treatment with inhibitors capable of blocking several upstream events leading to caspase activation. Cell cultures were treated with SB or co-treated with different dosages/durations of vitamin C, vitamin E, cyclosporine A and salubrinal (Sal), which can counter oxidative stress [33], lipid peroxidation [34], cytochrome C release [35] and EIF2α dephosphorylation [36], respectively. Lysates from these cultures were compared for α-Syn oligomer accumulation. Among the agents tested, Sal was the only one capable of preventing the SB-treated cells from both apoptosis and accumulation of α-Syn aggregates (Figure 8); hence, ER stress response became the focus of our subsequent studies. As reported before, Sal treatment alone had no adverse effects on 3D5 cells.

To determine whether ER stress plays a role in α-Syn oligomer accumulation, we used a known ER stress inducer, tunicamycin (TM). Similar to SB-treated cells, the TM-treated cultures displayed higher levels of α-Syn oligomer than their non-treated counterparts (Figure 8B). Moreover, the increase was impeded by co-treatment with Sal, thus establishing a close link between ER stress and α-Syn oligomer accumulation.

To verify the effects of drug treatment on ER stress, lysates from α-Syn expressing 3D5 cells (marked as α-Syn+ in Figure 8), treated with or without SB, were probed with antibodies to several markers relevant to ER stress response (i.e. GRP78, p-EIF2α, XBP-1, ATF6α and CHOP) and with antibodies to GAPDH to serve as loading control (Figure 8B). The SB exposure caused an increase in the ratio of p-EIF2α to total EIF2α and that of CHOP and other ER stress markers to GAPDH (Additional file 4, compared SB to Con). α-Syn+ cultures co-treated with Sal and SB displayed lower levels of CHOP (a molecule involved in ER stress-induced apoptosis), XBP-1 (Additional file 4), but higher levels of p-EIF2α and GRP78 when compared to the SB-treated. The results are consistent with those reported previously, indicating that Sal is an inhibitor of EIF2α dephosphorylation and that such inhibition leads to an increase in molecular chaperones such as GRP78 and a decrease in CHOP [37-39]. Studies of 3D5 cultures exposed to other HDAC inhibitors (i.e. Agk2, VPA) yielded comparable results (Additional files 5 &6).

### Overexpression of α-Syn elicits ER stress response

Previous studies reported that induced expression of A53T mutant α-Syn led to PC12 cell death and that the toxicity is mediated via ER stress and mitochondrial cell death pathways [40]. However, it was unknown whether induced expression of wild-type α-Syn in 3D5 cells also elicits an ER stress response. We investigated this issue by probing 3D5 cell lysates with or without induced α-Syn expression for ER stress markers. The induced cultures (α-Syn+) displayed a higher ratio of either GRP78 to GAPDH or p-EIF2α to total EIF2α than their non-induced (α-Syn-) counterparts, indicating the overexpression of wild-type α-Syn *per se *can cause an ER stress response (Figure 8B & Additional file 4). It is worth noting that without drug treatment neither α-Syn- nor α-Syn+ cultures had detectable amounts of CHOP, XBP-1 or ATF6α.

### Induced expression of α-Syn sensitizes 3D5 cultures to SB or TM toxicity via ER stress response

Cultures from α-Syn+ and α-Syn- were either treated with SB, TM or not for 36 hrs followed by cell viability assay (Figure 8A). At this time point cell death occurred in both cultures with drug treatment. The drug exposure significantly caused more cell loss in the α-Syn+ cultures than their α-Syn- counterparts. This difference in drug response was significantly reduced in cultures exposed to SB plus Sal or to TM plus Sal.

Similar to what we observed in α-Syn+ cultures, exposure of α-Syn- cultures to SB, VPA, Agk2 or TM caused an increase in ER stress response (Figure 8B, Additional files 4 &5). However, the drug treatment was less effective in altering the level of most ER stress markers and activation of caspase 3 in α-Syn- than α-Syn+ cultures. Together, the results indicate that α-Syn overexpression elicits ER stress response leading to the sensitization of 3D5 cultures to insults.

α-Syn- cultures co-treated with Sal and SB or other aforementioned agents also led to increase in the level of EIF2α phosphorylation and that of GRP78. However, the amount of ATF6 was not affected. The result is consistent with a recent report in which Sal treatment did not affect ATF6 ER stress pathway [41].

### ER stress response, α-Syn aggregation and apoptosis in primary neuronal cultures exposed to SB and other HDAC inhibitors

To determine whether the effects of HDAC inhibitors treatment on α-Syn aggregation and ER stress response are unique to 3D5 cultures, we next compared primary cultures from α-Syn PAC mice (also referred to as TG mice) to those of non-transgenic (NT) littermates for their responses to the drug exposure, focusing first on cell viability. The TG mice expressed low levels of wild-type human α-Syn and did not have α-Syn aggregates or neurodegeneration at any age (up to 24 months).

**Figure 9:**
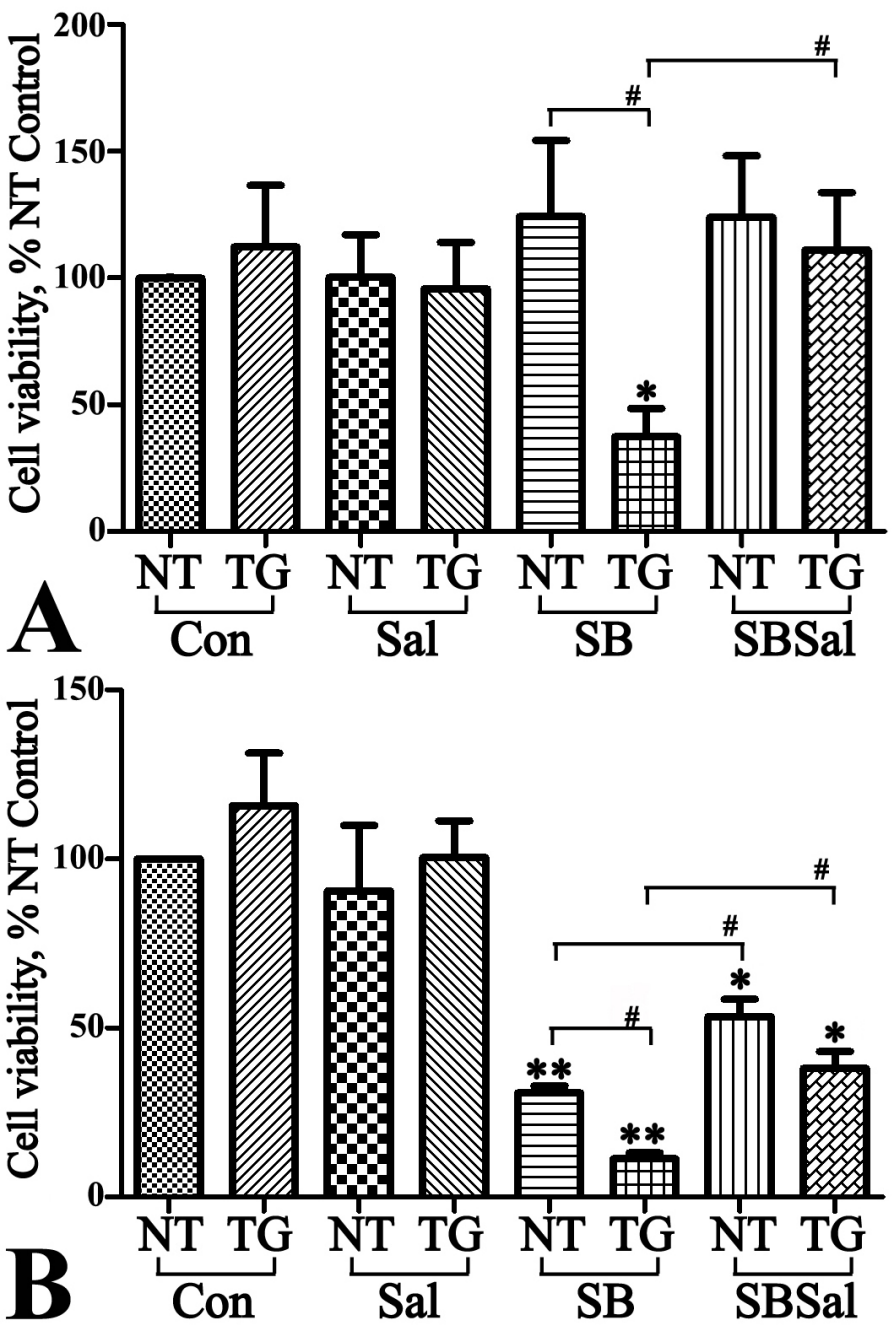
**Effects of SB and salubrinal on the survival of primary neuronal cultures**. Primary neuronal cultures from TG and NT mice were exposed to 10 mM SB, 20 μM Sal, SB plus Sal for **(A) **2 days and **(B) **4 days, Non-treated counterparts were included as controls (Con). Without any drug treatment, cultures from TG and NT mice have a similar number of cells. A 2-d treatment of cultures with SB did not affect the survival of NT cultures, but resulted in a significant (*P < 0.05) cell loss in TG cultures when compared to untreated counterparts. The effect of SB on TG cell survival was significantly (#P < 0.05) blocked by co-treatment with Sal. Cell loss was noted in NT cultures treated with SB for 4 ds (**P < 0.01), but the extent of such change is significantly (#P < 0.05) less than that demonstrated in TG cultures with same duration of SB treatment. Co-treatment with Sal also reduced the cell loss caused by a 4d exposure to SB.

Cultures from both TG and NT mice had similar cell numbers before their exposure to the drug. After 2 ds of SB exposure (Figure 9A), significant cell loss was demonstrated in TG cultures whereas such cell loss in NT cultures was detected after 4 ds of drug treatment (Figure 9B). In comparison, a significant cell loss was observed after 24 hrs of the drug exposure in SB-treated 3D5 cultures. Consistent with what we observed in 3D5 cultures, co-treatment with Sal significantly protected primary neuronal cultures from the cytotoxicity of SB (Figure 9) or Agk2 (Additional file 7).

**Figure 10:**
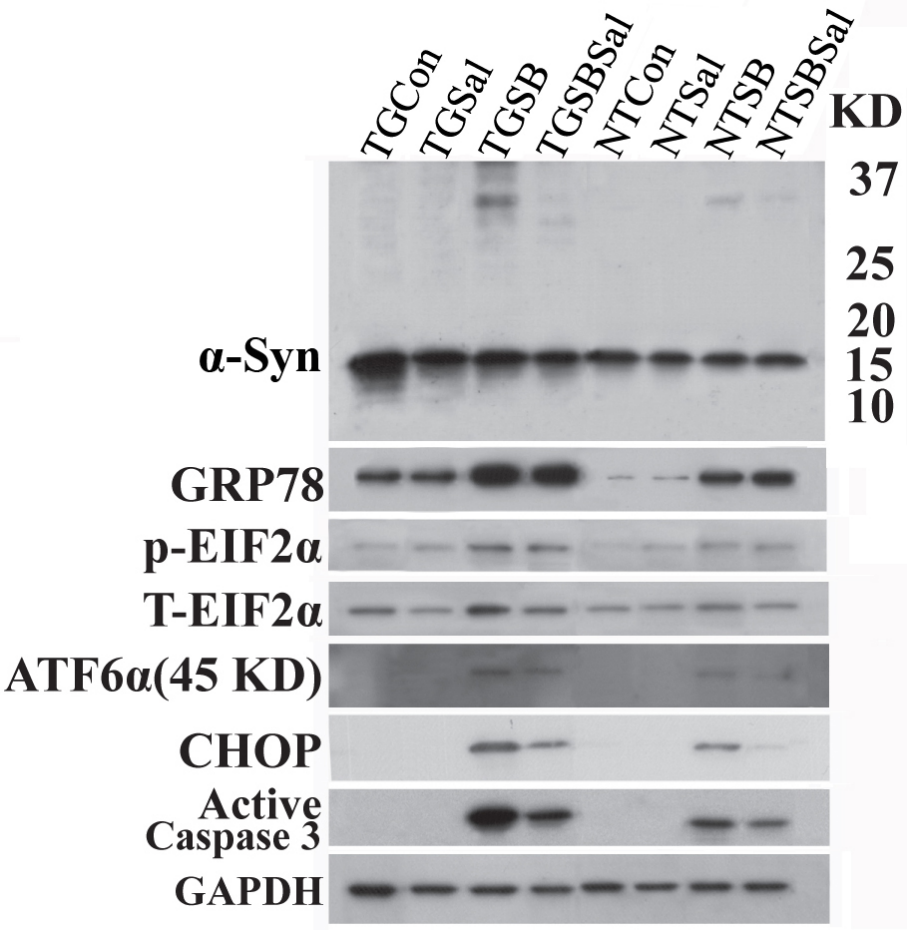
**SB and Sal exposure of primary neuronal cultures affects ER stress response, apoptosis and α-Syn aggregation**. Primary neuronal cells from TG and NT mice, after 7 days in cultures, were treated with 10 mM SB or SB plus 20 μM Sal for 96 hs. Non-treated counterparts were included as controls (Con). Proteins extracted from different cultures were evaluated by Western blotting using antibodies to five ER stress markers, active caspase 3, α-Syn and GAPDH. Cells from TG mice expressed about 1.6 times (normalized to GAPDH) more α-Syn than those from NT controls. The SB-treated TG and NT cultures (TGSB & NTSB) expressed more abundant ER stress markers than their controls, and such stress response and caspase 3 activation are greater for the TG than NT cultures. These changes were reduced in cultures treated with SB plus Sal. The results are consistent with those obtained from the 3D5 cell line, which expresses higher α-Syn than primary neuronal cultures.

Overexpression of α-Syn in TG specimen was confirmed by Western blot analysis of cell lysates from primary cultures. The level of α-Syn monomers in TG cultures is about 1.6 times of that detected in NT controls (Figure 10, compared lanes 1 and 5). In the absence of SB exposure, α-Syn oligomers were not readily detected in TG or NT cultures. Upon SB treatment, both cultures accumulated α-Syn of a size consistent with that of dimer (i.e. ~34 kDa), but the amount appeared to be greater for the TG cultures. The increase in α-Syn dimer did not result in the generation of inclusions detectable by immunocytochemical staining with α-Syn antibodies or Thioflavin S labeling. Co-treatment of primary cultures with SB and Sal resulted in reduced level of α-Syn dimers. The results are consistent with those demonstrated in 3D5 cultures.

Treatment of primary neuronal cultures with SB also resulted in an ER stress response and activation of caspase 3 (Figure 10). Importantly, such response was more intense in the SB-treated TG cultures than similarly treated NT controls and less intense with Sal co-treatment (Additional file 8).

## Discussion

Many studies have been devoted to identifying the mechanisms involved in α-Syn assembly and determine whether the accumulation of α-Syn assemblies is cytotoxic or protective. Although the results are far from conclusive, it is reasonable to suggest that the physicochemical property of α-Syn oligomers determines whether they are cytotoxic and what relation the resultant cytotoxicity has to aggregate formation. In this regard, the 3D5 transfectant is of great value for identification of pathways that modulate the risk of α-Syn-mediated cytotoxicity and agents capable of inhibiting accumulation of α-Syn and its assemblies, due to its ability to accumulate α-Syn assemblies without compromising cell survival [14].

A number of agents have been tested in previous cell-based studies and shown to reduce α-Syn aggregation and its toxicity [11,12,42,43]. In the present study, we investigated the effect of SB, Val or Agk2 treatment on α-Syn assembly/accumulation in 3D5 cultures. We demonstrated that exposure to each of these drugs reduced the viability of differentiated, α-Syn induced 3D5 cells and enhanced α-Syn oligomer accumulation, and that overexpression of wild-type α-Syn alone is not cytotoxic. This is in contrast to what was observed in previous studies [12], in which overexpression of α-Syn in the nuclei, but not cytoplasm, of neuroblastoma cells, SH-SY5Y, resulted in cell death. Treatment of SH-SY5Y cultures with 10 mM SB for 48 hs was shown to protect cells from toxicity caused by nuclear accumulation of α-Syn, but did not adversely affect those with cytoplasmic α-Syn accumulation. Treatment of H4 cells overexpressing A53T α-Syn with Agk2 was shown to reduce cell death caused by cytoplasmic α-Syn aggregates. In contrast, treatment of α-Syn overexpressing 3D5 cells with Agk2 caused cell death. The discrepancy may reflect differences in cell type, as supported by the results from our analysis of non-induced 3D5 cultures and non-transfected SH-SY5Y cells (Additional file 9). Cell death was noted in 3D5 cultures within 24 hs of SB (10 mM) treatment. In comparison, cell death was not observed up to 48 hrs after treatment of SH-SY5Y cells, indicating they are less sensitive than 3D5 cells to SB cytotoxicity. Therefore, to evaluate whether drug treatment has beneficial effects or not in cell-based studies, it is important to examine more than one cell type. For this reason, we included primary neuronal cultures in the present studies. This also allowed us to substantiate the pathobiological relevance of findings from cell-line based studies.

We showed that exposing 3D5 and primary cultures to HDAC inhibitors caused an accumulation of α-Syn oligomers, an increase in ER stress response and a decrease in cell survival. In this regard, several recent studies have demonstrated the induction of ER stress response in cell cultures following treatment with different HDAC inhibitors [30-32]. In our studies, such stress response was greater for cultures with α-Syn overexpression than those without. We considered that the difference is at least in part, if not entirely, due to differences in the basal level of ER stress markers. This is in light of our results that (i) α-Syn overexpression alone, regardless of whether it led to the assembly of filamentous α-Syn [14] or not, elicited ER stress response, (ii) inhibition of EIF2α dephosphorylation via Sal treatment protected cultures from undergoing apoptosis caused by HDAC inhibitors exposure, (iii) treatment of cultures with ER stress inducer TM also resulted in α-Syn oligomer accumulation as in the HDAC inhibitors treated, and (iv) such accumulation was reduced by co-treatment of cultures with Sal. It has been reported that overexpression of A53T mutant α-Syn or wild-type α-Syn phosphorylated at serine 129 caused ER stress and cell death in PC12 or SH-SY5Y cells [40,44], and that expression of human α-Syn in yeast cells led to blocking of ER-Golgi traffic [45]. Recently, induced α-Syn expression was shown to increase the vulnerability of PC12 cells to stressors such as thapsigargin, TM and 1-methyl-4-phenyl-pyrisinium (MPP+) [46]. We also have data demonstrating differences between the induced and non-induced 3D5 cultures in sensitivity to neurotoxin treatment (i.e. MPP+, rotenone, 6-hydroxydopamine(6-OHDA), paraquat) (Additional file 10). Other investigators have also provided evidence supporting the important role of ER stress response in PD pathogenesis. For example: (i) activation of ER stress response was detected in dopaminergic neurons in the substantia nigra of postmortem PD cases [47], and (ii) PD animal or cell models generated via exposure to neurotoxins displayed ER stress response [48,49].

We do not know how HDAC inhibitor treatment leads to ER stress in neuronal cells. However, recent studies of non-neuronal cells demonstrated that (i) exposure of breast cancer cells to panobinostat (a pan HDAC inhibitor) led to deacetylation of GRP78, dissociation of GRP78 from PERK and increase the levels of pEIF2α, ATF4 and CHOP [32], (ii) exposure of colorectal carcinoma cells to tricostatin A (a pan-HDAC inhibitor) affected the binding of HDAC to GRP78 promotor [30] and (iii) the C-terminal region of RTN-C protein, an ER membrane protein, can interact with HDAC8 *in vitro *[50].

Our studies showed that 3D5 and primary cultures have different sensitivities to HDAC inhibitor treatment. When the same dosage of SB or VPA was used to treat these cultures, it required a longer duration of incubation for primary cultures to display cytotoxicity. In contrast, 3D5 cells were less sensitive than primary cultures to Agk2 exposure. The cause(s) of such differences in drug sensitivity remains unknown. We noted that a previous study has demonstrated a protective effect of Agk2 treatment on rat primary neuronal cultures overexpressing A53T α-Syn via lentiviral infection of constructs containing mutant α-Syn [11]. The discrepancy between the previous study and our primary neuronal culture study may be due to differences in experimental paradigm. Low levels of overexpressing wild-type human α-Syn in mice had no impact on the viability of mouse primary neuronal cultures. In comparison, overexpression of A53T α-Syn by viral infection reduced cell survival.

We showed that 3D5 and primary cultures treated with HDAC inhibitors have different quantity/quality of α-Syn oligomers. Most α-Syn oligomers appearing in primary neuronal cultures are dimers and relatively soluble, whereas those in 3D5 cells are larger oligomers with different solubility (ranging from buffer-extractable to sarkosyl-insoluble). The distinction may reflect difference in α-Syn expression levels. This is supported by our findings that the level of α-Syn in TetOff induced 3D5 cells is far greater than that expressed in primary neuronal cultures from TG mice (based on equal sample loading (Additional file 11). Moreover, it has been reported that a critical concentration of α-Syn is required to initiate α-Syn assembly in test tubes, and that formation of dimer is an early step of α-Syn assembly [51]. The difference between cell types in α-Syn oligomers production may also reflect the rate of α-Syn assembly. In this regard, several molecules have been shown to promote α-Syn assembly *in vitro *and *in vivo *[52-56]. It is possible that the amount/type of endogenous inducer for α-Syn assembly may vary in different cell types.

Our finding that an overexpression of wild-type human α-Syn in both 3D5 and primary cultures can elicit ER stress response is noteworthy. According to some studies, the accumulation of α-Syn in the human brain elevates significantly during aging [57,58]. There is also ample evidence that the unfold protein response is compromised with aging [59]. Therefore, there is a potential risk of using HDAC inhibitors as therapeutics for elderly subjects with neurodegenerative disorders [60].

## Materials and methods

### Conditional transfectant 3D5

Alpha-Syn transfectant 3D5 was derived from a human neuroblastoma cell line (BE2-M17D) and characterized previously [13,14]. The transfectant expresses wild-type human α-Syn and displays neuronal phenotypes upon TetOff induction and incubation with RA [14]. Cultures of 3D5 were maintained in DMEM/10% fetal bovine serum with 2 μg/mL Tet (referred to as non-induced or α-Syn-) or without (referred to as induced or α-Syn+) at 37°C and 5% CO_2_. Those intended for biochemical analysis were seeded at 0.5 × 10^6 ^cells/plate (100 × 20 mm, BD Biosciences, San Jose, CA) or 1.5 × 10^5 ^cells/well in 6-well plates. For immunofluorescence or spectrophotometric assay, 3D5 cells were seeded at 2 × 10^4 ^cells/well on coverslips in 24-well plates and 1 × 10^4 ^in 48-well plates (Bellco Glass Inc, Vineland, NJ), respectively. Media were replaced the next day with Neurobasal medium (Invitrogen, Carlsbad, CA), 2% B-27 supplement (antioxidant free, Invitrogen), 2 mM L-glutamine (Sigma) and 10 μM RA (Sigma-Aldrich, St Louis, MO). After 10 ds of differentiation and induced α-Syn overexpession, cells were treated with HDAC inhibitors [SB (sigma), VPA (Sigma) and Agk2 (EMD Biosciences, La Jolla, CA)], a derivative of short-chain fatty acid [sodium acetate (SA)], an endoplasmic reticulum (ER) stress inducer [tunicamycin (TM, Sigma)], an inhibitor of phosphatases that act on the eukaryotic translation factor 2 subunit alpha (EIF2α) [salubrinal (Sal, Tocris Bioscience, Ellisville, Missouri)], a protein synthesis inhibitor [cycoheximide (CHX, Sigma), or a pan caspase inhibitor (CI) [Z-VAD (OMe)-FMK (EMD Biosciences)]. The cultures were treated with different drug concentrations for durations described in the *Result *section. Some cultures were treated with two of the aforementioned drugs or with SB and tetracycline (2 μg/ml, for blocking α-Syn induction).

Primary neuronal cultures were prepared from hippocampal and cortical brain tissues of postnatal (day 1) mice (see below) with or without transgenic expression of wild-type human α-Syn, according to a protocol reported previously [61]. They were plated at 6 ~ 7 × 10^5 ^cells per well on poly-D-lysine (Sigma) coated 6-well plate and maintained for 7 ds before treatment in the absence or presence of different drugs for different durations.

### Animals

All animal procedures were approved by the Mayo Clinic Institutional Animal Care and Use Committee (IACUC) and were in accordance with the National Institute of Health Guide for the Care and Use of Laboratory Animals (NIH Publications No. 80-23) revised 1996.

### Alpha-Syn PAC transgenic mice

Mice were provided by Dr. Farrer's Laboratory and will be described in detail elsewhere. Briefly, transgenic mice were generated using a P1 artificial Chromosome (PAC) RP-1 27 M07, which was previously identified to contain the entire wild-type human α-Syn gene and regulatory sequences PAC DNA [62]. Purified DNA was injected into FVB/N (Taconic, Germantown, NY) fertilized oocytes and transplanted into pseudo-pregnant ICR (Harlan, Indianapolis, IN) female mice. Transgenic founders were genotyped by PCR and analyzed for gene expression by quantitative RT-PCR and western blotting. The founder line with the highest expression was expanded and maintained on FVB/N background (Taconic). Human protein expression in 6 month-old mice is approximately 2 fold of endogenous murine α-Syn. The expression level is highest in the hippocampus followed by cortex, olfactory tubercle and striatum.

### Antibodies

To detect α-Syn, we used monoclonal antibody Synuclein-1 (BD Biosciences) and polyclonal antibody Ab98 [63]. For other proteins we used rabbit polyclonal antibodies to total and cleaved Caspase-3, Caspase 9 and PARP (Cell Signaling, Danvers, MA), phosphorylated EIF2α (pEIF2α, Invitrogen), GADD153/CHOP10 (Sigma), XBP-1 and ATF6α (Santa Cruz Biotechnology, Santa Cruz, CA). We also used monoclonal antibodies to GRP78 (BD Biosciences), total EIF2α (T-EIF2α, Cell Signaling) and GAPDH (1D4, Covance Research Products, Berkeley, CA). The binding of primary antibody to protein of interest was detected using peroxidase-conjugated goat anti-rabbit or anti-mouse IgG antibodies (Chemicon, Temecula, CA) and Alexa594 or Alexa488 goat anti-mouse antibodies (Invitrogen).

### Western blot analysis

Cell cultures were harvested and centrifuged at 200 × g for 15 min. The pellet was resuspended in a lysis buffer [20 mM MES, pH 6.8; 80 mM NaCl, 1 mM MgCl_2_, 2 mM EGTA, 10 mM NaH_2_PO_4_, 20 mM NaF, phenylmethylsulfonyl fluoride (PMSF, 1 μg/ml and leupeptin, 10 μg/mL], homogenized and centrifuged at 180 × g for 15 min at 4°C. The cell lystates were mixed with Tricine-SDS sample buffer (Invitrogen) and 2% β-mercaptoethanol, boiled for 5 min and resolved by SDS-PAGE using 10-20% Tris/Tricine gel (Bio-rad, Hercules, CA). Precision plus protein standards (Bio-Rad) were included as references. Proteins separated by SDS-PAGE were transferred onto nitrocellulose and processed for immunolabeling for proteins of interest. Membranes probed with GAPDH antibody were used as loading controls. Immunoreactivity was visualized with enhanced chemiluminescence (ECL plus, Amersham Pharmacia Biotech, Buckinghamshire, UK) or SuperSignal West Femto Maximum Sensitivity Substrate (Thermo Scientific, Rockford, IL) and analyzed as before [64].

### Fractionation Studies of Culture Specimens

Cell lysates were fractionated to derive buffer extractable (SN1), 5 M NaCl/1% sarkosyl-soluble (SN2) and 1% sarkosyl-insoluble fractions (SKI) as reported [14]. The SKI fraction was finally resuspended in 50 mM Tris (pH 8.0). All fractions and lysates were analyzed by Western blotting. A portion of the SKI sample from TetOff induced cultures with or without SB treatment and corresponding sample from cultures without α-Syn induction were analyzed further (see below).

### Electron and immunoelectron microscopies

Sakosyl-insoluble samples were processed for electron and immunoelectron microscopies using protocols described previously [64]. Cultures with or without SB treatment were processed as before [14] for electron microscopy.

### Immunocytochemistry

Cells grown on coverslips were rinsed with PBS, fixed in 4% paraformaldehyde and permeabilized with 0.1 M Tris-buffered saline (TBS, pH 7.6) containing 0.5% triton X-100 for 5 min. They were subsequently blocked with 3% goat serum in TBS, incubated with antibody Synuclein-1 in TBS containing 1% goat serum overnight at 4°C then incubated with Alexa594 goat anti-mouse secondary antibody for 1 h. For Thioflavin S staining the coverslips were immersed in 0.0005% thioflavin S/PBS/formalin and washed 3 times with 70% ethanol and TBS. After immuno- and thiofavin S staining, the coverslips were stained with DAPI (Invitrogen) for 10 min to locate the nuclei and evaluated by confocal fluorescence microscopy (Zeiss LSM 510; Carl Zeiss MicroImaging, Thornwood, NY).

### Cell viability

The viability of 3D5 cells was assessed by incubation of cultures with 2 μM calcein AM (Invitrogen), according to the manufacturer's recommendation. Fluorescence emitted from live cells was measured at 495 nm (excitation)/530 nm (emission) using a Cary Eclipse Fluorometer and Spectra Max M2 and Soft Max Protein 4.6 software (Molecular Devices, Sunnyvale, CA). All measurements were performed in triplicate. Viability of primary neuronal cultures was assessed by counting cells in live cultures. We randomly captured the image of cultures at 10-15 fields (200×). Each image was evaluated independently by two-individuals for intact cells to derive the number of cells in all fields as well as the average cell counts per field. After image capturing, the cultures were processed for Western blot analysis.

### Statistical analysis

Data from at least 3 sets of independent experiments were analyzed by one-way Anova with Dunnett's *post hoc *test or Student's t test to determine statistical significance.

## List of Abbreviations

α-Syn: alpha-synuclein; CHX: cycloheximide; CI: caspase inhibitor; ER: endoplasmic reticulum; HDAC: histone deacetylase; NT: non-transgenic; RA: retinoic acid; Sal: salubrinal; SKI: sarkosyl-insoluble; SA: sodium acetate; SB: sodium butyrate; SN1: buffer-soluble, SN2: salt plus sarkosyl-extractable; α-Syn+: cultures with induced α-Syn expression; α-Syn-: cultures without induced α-Syn expression; TG: transgenic; TetOff: tetracycline-off, TL: total lysates; TM: tunicamycin; VPA: valproic acid;

## Competing interests

The authors declare that they have no competing interests.

## Authors' contributions

PJ designed the study, executed most of the experiments and analyzed the data. GM executed some of Western blots and involved in data analysis. ABE maintained animals and involved in the preparation of primary neuronal cultures. W-LL performed ultrastructural analysis. HM provided transgenic animals. SHY oversaw the study, analyzed the data and wrote the paper. All authors have read and approved the final manuscript.

## Supplementary Material

Additional file 1 **Effects of Agk2 and VPA exposure on α-Syn oligomerization and caspase 3 activation**. 3D5 cells with 10 days (ds) of RA elicited differentiation and TetOff-induced α-Syn expression were exposed to Agk2 (21 μM) and VPA (10 mM) for 18 and 36 hours (hs). Cultures without the drug treatment for 36 hs were used as controls (Con). Proteins from these cultures were resolved by SDS-PAGE and probed with antibodies to α-Syn, GAPDH and active caspase 3. The Agk2 and VPA treated cells contained more α-Syn oligomers (34 to 230 kDa in size) and cleaved caspase 3 than non-treated counterparts. The results are similar to those obtained from cultures treated with sodium butyrate. Molecular weight markers shown in current or subsequent figures were used to calibrate the size of α-Syn products. Asterisk marks monomeric α-Syn. Click here for file

Additional file 2 **Distinct differences between sodium acetate (SA) and sodium butyrate (SB) exposure on α-Syn oligomerization**. 3D5 cells with 10 days (ds) of RA elicited differentiation and TetOff-induced α-Syn expression were treated with SA (20 mM), SB (10 mM) or no drugs (Con) for 24 hs. Proteins from lysates of these cultures were resolved by SDS-PAGE and probed with antibodies to α-Syn and GAPDH. In contrast to that demonstrated in the SB-treated cultures, SA treatment did not lead to an increase of α-Syn oligomers. Asterisk marks monomeric α-Syn. Click here for file

Additional file 3 **Inhibition of α-Syn expression at the transcription or translation level minimally affected SB induced α-Syn aggregation in 3D5 cells with 10 ds of differentiation and induced α-Syn expression**. Cultures were exposed to SB (10 mM), SB plus 2 μg/ml Tet (to block α-Syn induction) or SB plus cycloheximide (CHX, 20 μM, to inhibit protein synthesis) for 36 hs. Cultures without any drug treatment or treated with Tet or CHX only were included as controls. Cell lysates were probed with antibodies to α-Syn and GAPDH. Cultures co-treated with SB and Tet or SB and CHX contained more α-Syn oligomers than those without drug treatment or treated with Tet or CHX alone. The co-treated cultures contained α-Syn oligomers slightly less than those treated with SB, indicating the accumulation of α-Syn oligomers in SB-treated cells is unlikely caused by an increase of α-Syn expression. Click here for file

Additional file 4 **Quantification of Western blot shown in Figure **8 Click here for file

Additional file 5 **Effects of VPA, Agk2 and co-treatment with Sal on accumulation of α-Syn oligomers and expression of ER stress markers**. Differentiated 3D5 cells with induced α-Syn expression (α-Syn+) or without the induction (α-Syn-) were treated with Sal (40 μM), VPA (10 mM), VPA plus Sal (VPASal), Agk2 (21 μM) or Agk2 plus Sal (Agk2Sal) for 24 hs. Cells without the drug treatment were included as controls (Con). Western blotting demonstrated that VPA or Akg2 exposure leads to a marked increase of α-Syn oligomers, ER stress markers CHOP and p-EIF2α/T-EIF2α and a moderate increase of GRP78 in the α-Syn + cells. Without any drug treatment, GRP78 was detected more in the α-Syn+ than the α-Syn- cultures. Click here for file

Additional file 6 **Quantification of Western blot shown in additional file **5 Click here for file

Additional file 7 **Survival of TG or NT primary neuronal cultures exposed to Agk2, Sal and both**. Primary cultures TG and NT mice were treated with Agk2 (7 μM), Sal (20 μM) or Agk2 plus Sal (Agk2Sal) for 24 hs. TG cultures were more sensitive to Agk2 treatment than NT controls, and co-treatment with Sal can protect cultures from Agk2 toxicity. *P < 0.05, compared TGCon to TGAgk2; #P < 0.05, compared TGAgk2Sal to TGAgk2 or NTAgk2 to TGAgk2. Click here for file

Additional file 8 **Quantification of Western blot shown in Figure **10 Click here for file

Additional file 9 **Differences between 3D5 and SH-SY5Y cells in sensitivity to SB treatment**. 3D5 and SH-SY5Y cells were seeded in 24 well plates at the same number per well. They were exposed to SB (10 mM) and assessed for cell viability after 24, 48 and 96 hs of the drug exposure. Cultures without the drug treatment were included as controls (Con). Click here for file

Additional file 10 **Differential effects of neurotoxin exposure on the survival of 3D5 cells**. Differentiated 3D5 cells with induced α-Syn expression (α-Syn+) or without (α-Syn-), were treated with 6-OHDA (400 μM), MPP+ (3 mM), Paraquat (1.5 mM) and Rotenone (60 μM) for 30 hs, then assessed for cell viability by Calcein AM assay. *P < 0.05, compared between α-Syn + and α-Syn- cultures. Click here for file

Additional file 11 **Comparison of α-Syn expression level between TG primary neurons and 3D5 cells**. TG primary neuronal cultures as well as RA-differentiated and TetOff induced 3D5 cells after 7 ds and 10 ds in cultures, respectively, were harvested. Subsequent to protein extraction and quantification, equal amount of proteins from each sample were resolved by SDS-PAGE and immunoblotted with antibody for α-Syn. Results showed that 3D5 cells contain more α-Syn than primary cultures. Click here for file
