# Supplementary figures and images for: ER stress response plays an important role in aggregation of α-synuclein
Source: Mol Neurodegener. 2010 Dec 13;5:56. doi: 10.1186/1750-1326-5-56 (PMC3016345; doi:10.1186/1750-1326-5-56)

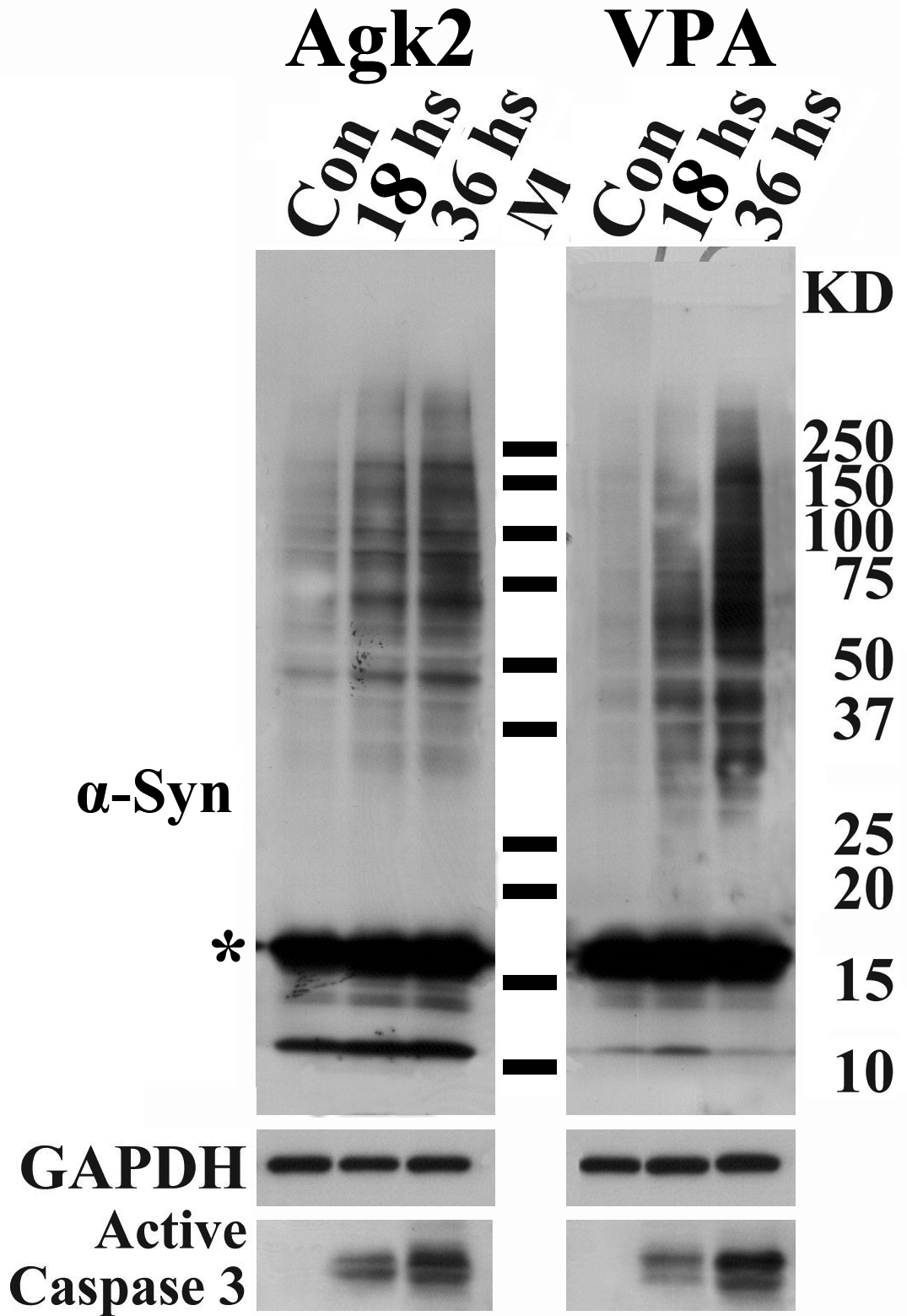

Supplement: Additional file 1 — Effects of Agk2 and VPA exposure on α-Syn oligomerization and caspase 3 activation. 3D5 cells with 10 days (ds) of RA elicited differentiation and TetOff-induced α-Syn expression were exposed to Agk2 (21 μM) and VPA (10 mM) for 18 and 36 hours (hs). Cultures without the drug treatment for 36 hs were used as controls (Con). Proteins from these cultures were resolved by SDS-PAGE and probed with antibodies to α-Syn, GAPDH and active caspase 3. The Agk2 and VPA treated cells contained more α-Syn oligomers (34 to 230 kDa in size) and cleaved caspase 3 than non-treated counterparts. The results are similar to those obtained from cultures treated with sodium butyrate. Molecular weight markers shown in current or subsequent figures were used to calibrate the size of α-Syn products. Asterisk marks monomeric α-Syn. [file 1750-1326-5-56-S1.JPEG]

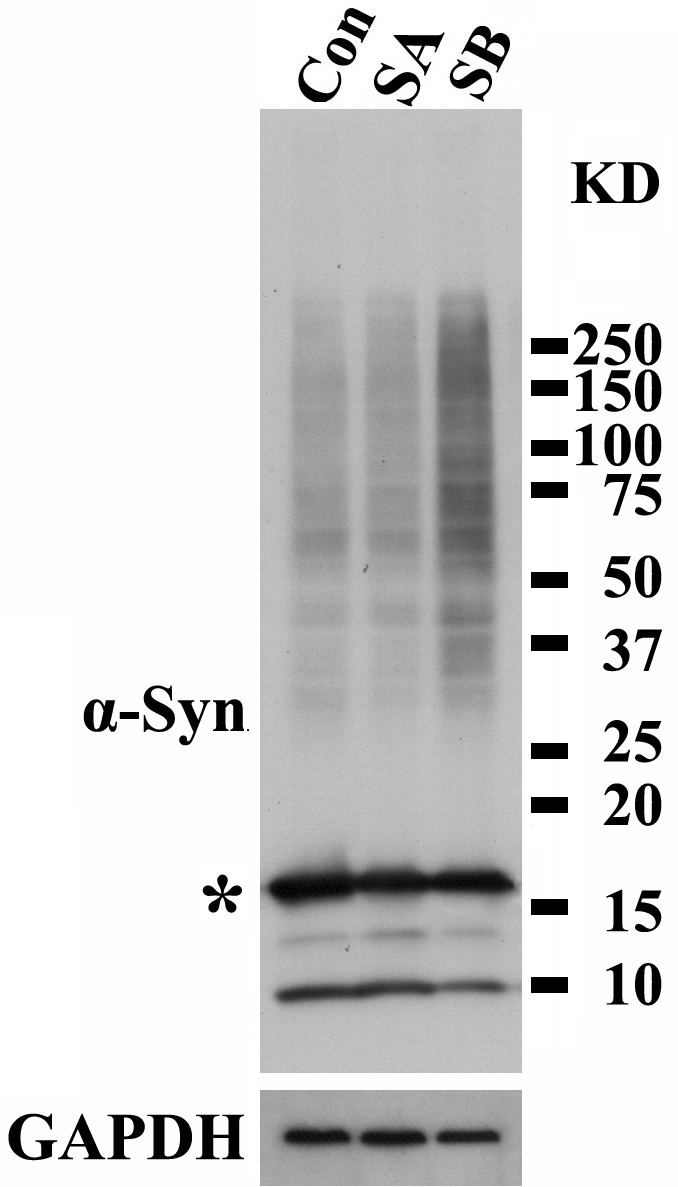

Supplement: Additional file 2 — Distinct differences between sodium acetate (SA) and sodium butyrate (SB) exposure on α-Syn oligomerization. 3D5 cells with 10 days (ds) of RA elicited differentiation and TetOff-induced α-Syn expression were treated with SA (20 mM), SB (10 mM) or no drugs (Con) for 24 hs. Proteins from lysates of these cultures were resolved by SDS-PAGE and probed with antibodies to α-Syn and GAPDH. In contrast to that demonstrated in the SB-treated cultures, SA treatment did not lead to an increase of α-Syn oligomers. Asterisk marks monomeric α-Syn. [file 1750-1326-5-56-S2.JPEG]

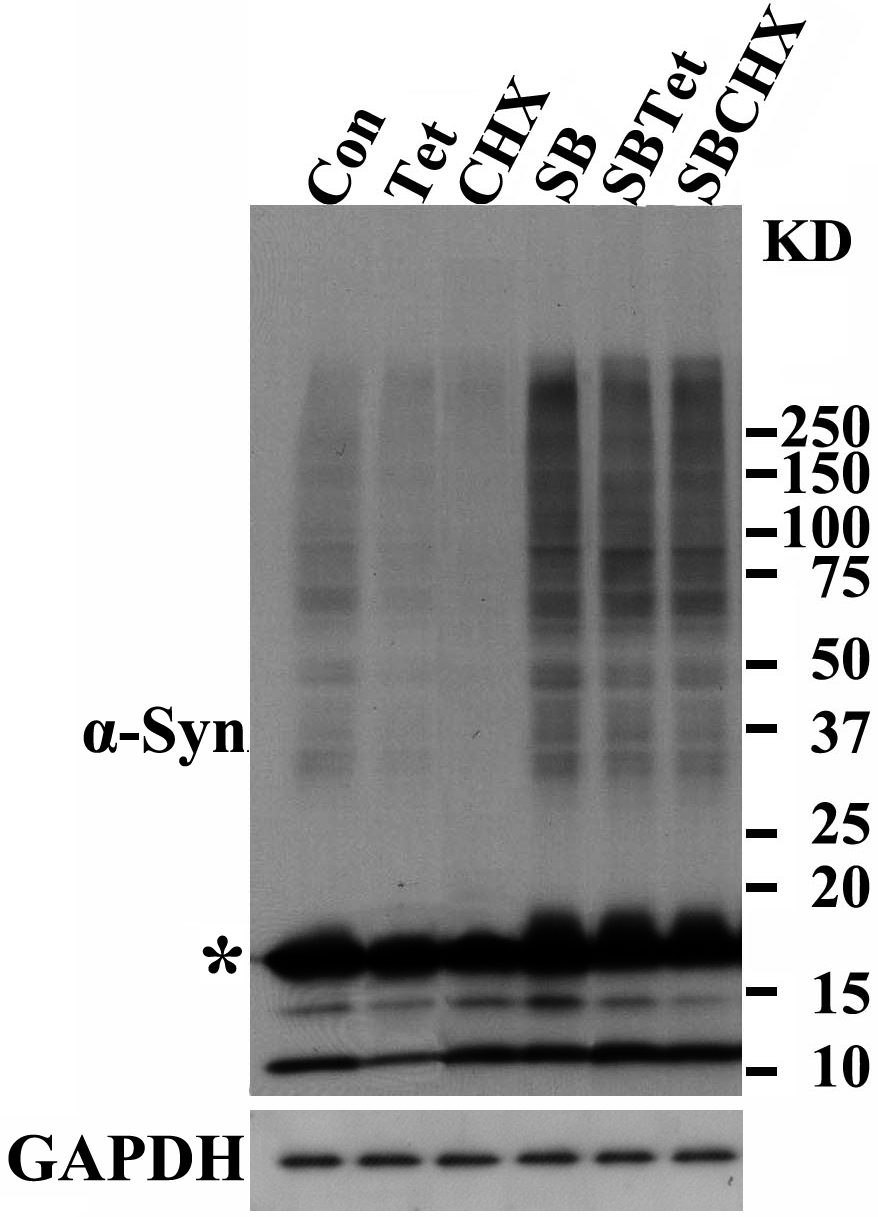

Supplement: Additional file 3 — Inhibition of α-Syn expression at the transcription or translation level minimally affected SB induced α-Syn aggregation in 3D5 cells with 10 ds of differentiation and induced α-Syn expression. Cultures were exposed to SB (10 mM), SB plus 2 μg/ml Tet (to block α-Syn induction) or SB plus cycloheximide (CHX, 20 μM, to inhibit protein synthesis) for 36 hs. Cultures without any drug treatment or treated with Tet or CHX only were included as controls. Cell lysates were probed with antibodies to α-Syn and GAPDH. Cultures co-treated with SB and Tet or SB and CHX contained more α-Syn oligomers than those without drug treatment or treated with Tet or CHX alone. The co-treated cultures contained α-Syn oligomers slightly less than those treated with SB, indicating the accumulation of α-Syn oligomers in SB-treated cells is unlikely caused by an increase of α-Syn expression. [file 1750-1326-5-56-S3.JPEG]

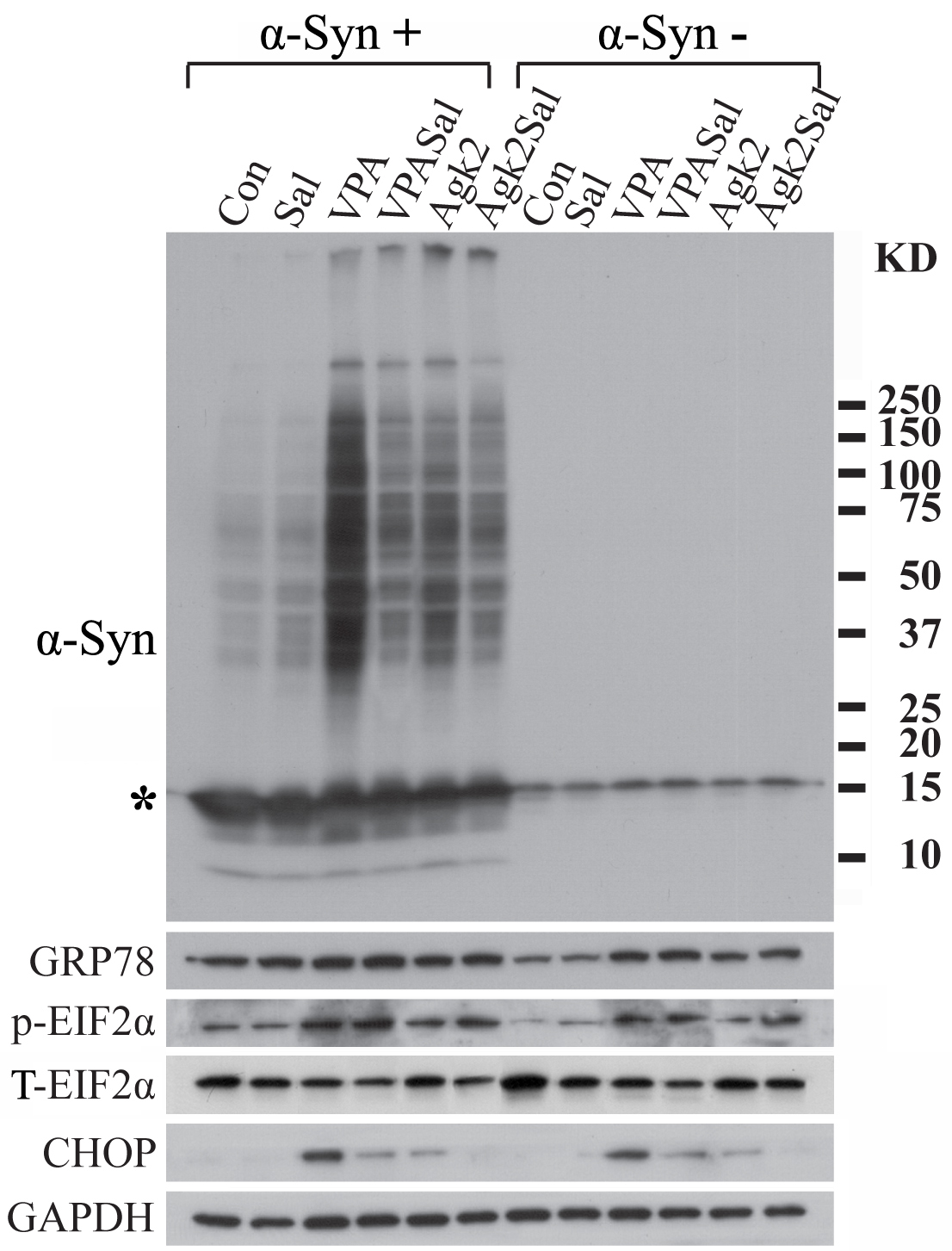

Supplement: Additional file 5 — Effects of VPA, Agk2 and co-treatment with Sal on accumulation of α-Syn oligomers and expression of ER stress markers. Differentiated 3D5 cells with induced α-Syn expression (α-Syn+) or without the induction (α-Syn-) were treated with Sal (40 μM), VPA (10 mM), VPA plus Sal (VPASal), Agk2 (21 μM) or Agk2 plus Sal (Agk2Sal) for 24 hs. Cells without the drug treatment were included as controls (Con). Western blotting demonstrated that VPA or Akg2 exposure leads to a marked increase of α-Syn oligomers, ER stress markers CHOP and p-EIF2α/T-EIF2α and a moderate increase of GRP78 in the α-Syn + cells. Without any drug treatment, GRP78 was detected more in the α-Syn+ than the α-Syn- cultures. [file 1750-1326-5-56-S5.JPEG]

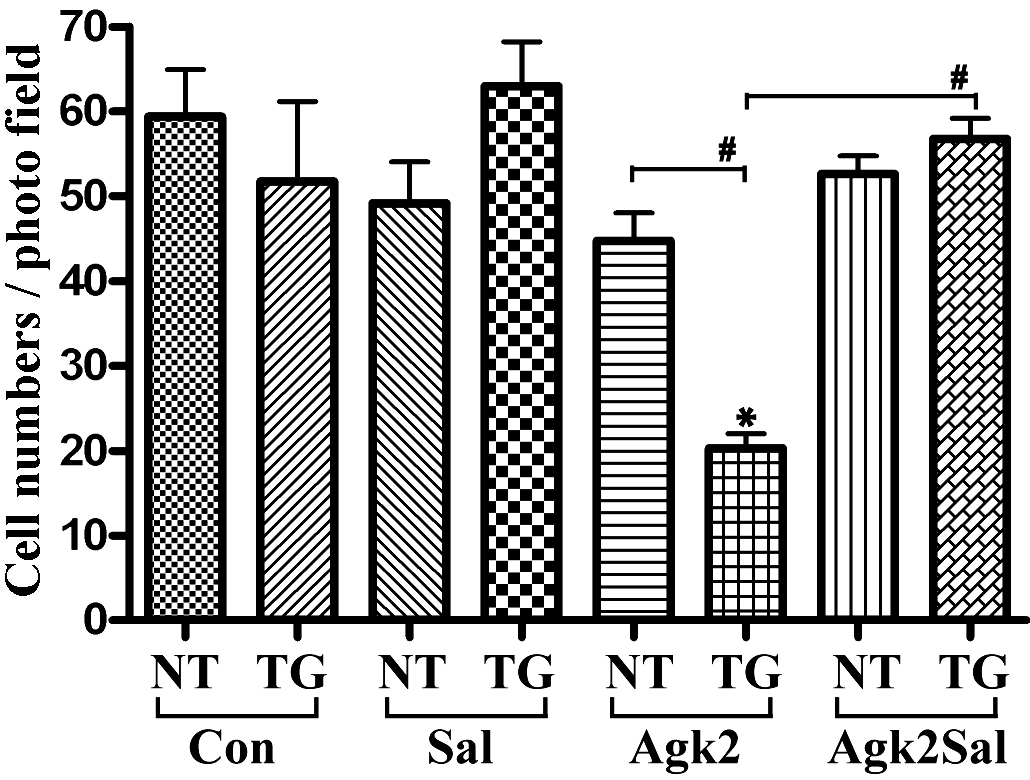

Supplement: Additional file 7 — Survival of TG or NT primary neuronal cultures exposed to Agk2, Sal and both. Primary cultures TG and NT mice were treated with Agk2 (7 μM), Sal (20 μM) or Agk2 plus Sal (Agk2Sal) for 24 hs. TG cultures were more sensitive to Agk2 treatment than NT controls, and co-treatment with Sal can protect cultures from Agk2 toxicity. *P < 0.05, compared TGCon to TGAgk2; #P < 0.05, compared TGAgk2Sal to TGAgk2 or NTAgk2 to TGAgk2. [file 1750-1326-5-56-S7.JPEG]

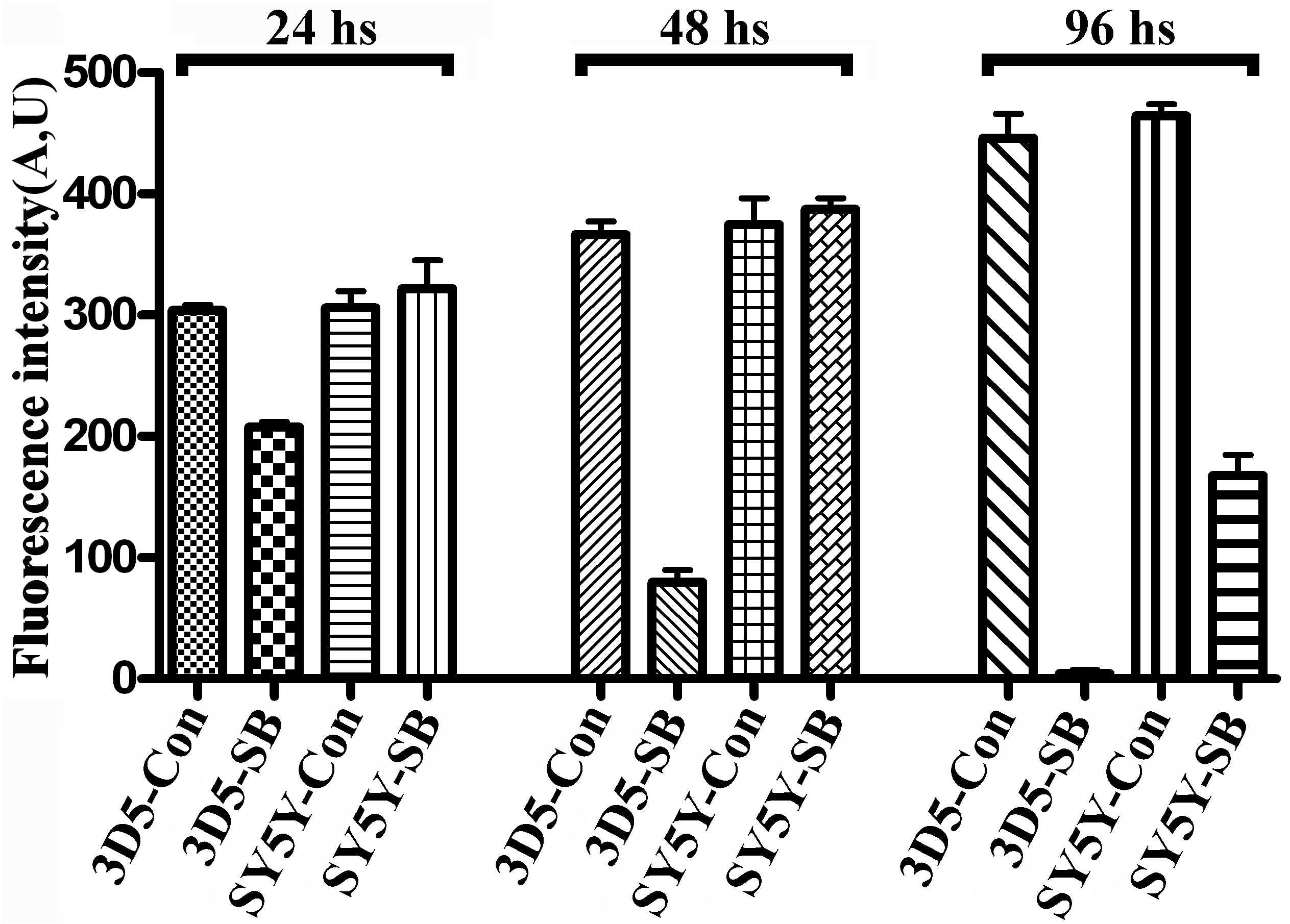

Supplement: Additional file 9 — Differences between 3D5 and SH-SY5Y cells in sensitivity to SB treatment. 3D5 and SH-SY5Y cells were seeded in 24 well plates at the same number per well. They were exposed to SB (10 mM) and assessed for cell viability after 24, 48 and 96 hs of the drug exposure. Cultures without the drug treatment were included as controls (Con). [file 1750-1326-5-56-S9.JPEG]

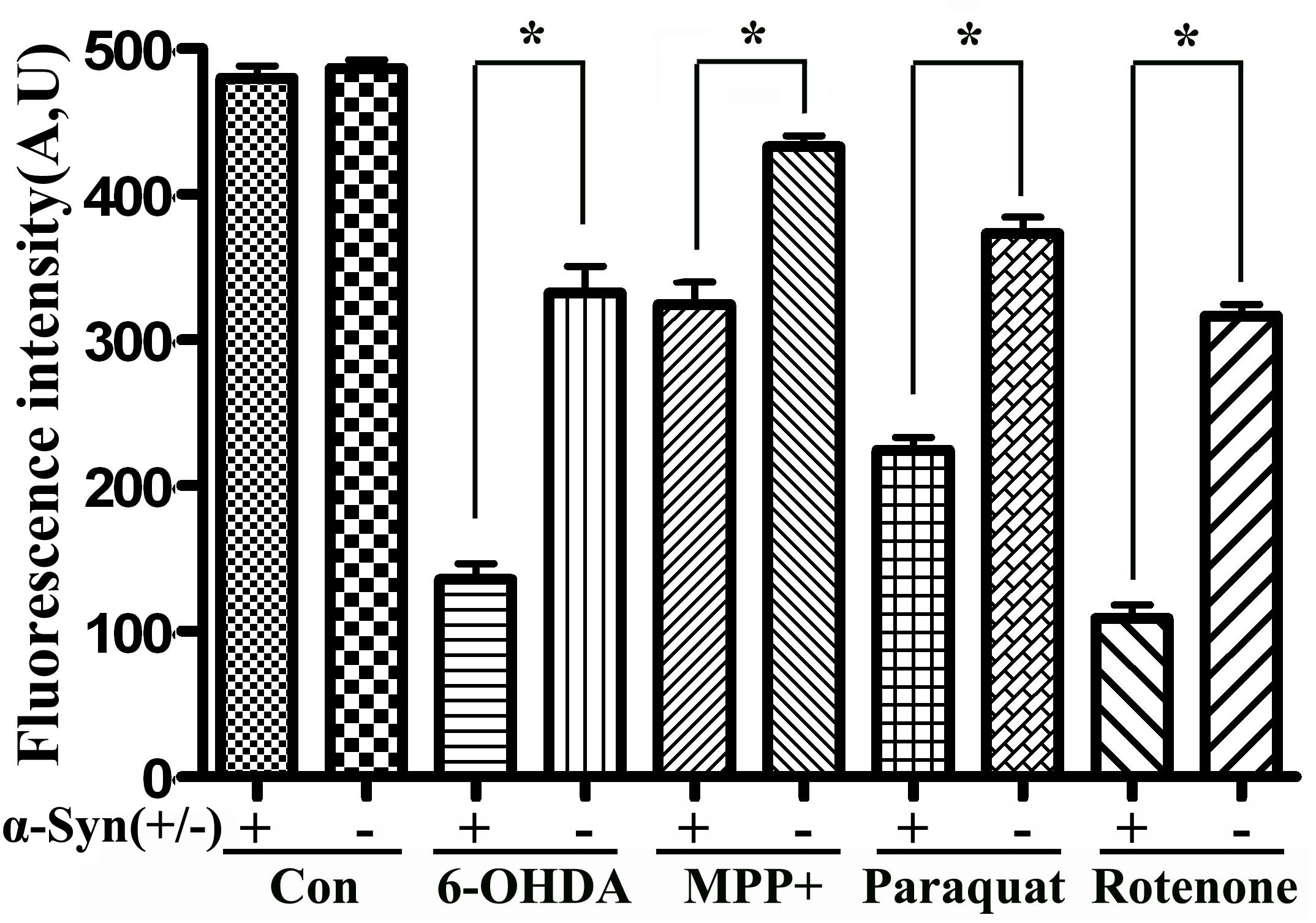

Supplement: Additional file 10 — Differential effects of neurotoxin exposure on the survival of 3D5 cells. Differentiated 3D5 cells with induced α-Syn expression (α-Syn+) or without (α-Syn-), were treated with 6-OHDA (400 μM), MPP+ (3 mM), Paraquat (1.5 mM) and Rotenone (60 μM) for 30 hs, then assessed for cell viability by Calcein AM assay. *P < 0.05, compared between α-Syn + and α-Syn- cultures. [file 1750-1326-5-56-S10.JPEG]

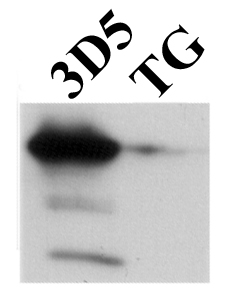

Supplement: Additional file 11 — Comparison of α-Syn expression level between TG primary neurons and 3D5 cells. TG primary neuronal cultures as well as RA-differentiated and TetOff induced 3D5 cells after 7 ds and 10 ds in cultures, respectively, were harvested. Subsequent to protein extraction and quantification, equal amount of proteins from each sample were resolved by SDS-PAGE and immunoblotted with antibody for α-Syn. Results showed that 3D5 cells contain more α-Syn than primary cultures. [file 1750-1326-5-56-S11.JPEG]
