# Supplementary material for: ER stress response plays an important role in aggregation of α-synuclein
Source: Mol Neurodegener. 2010 Dec 13;5:56. doi: 10.1186/1750-1326-5-56 (PMC3016345; doi:10.1186/1750-1326-5-56)
Supplement: Additional file 4 — Quantification of Western blot shown in Figure 8 [file 1750-1326-5-56-S4.DOC]

Additional file 4. Quantification of Western blot shown in Figure 8.

|  | α-Syn | EIF2α  P/Total | GRP78  GAPDH | CHOP  GAPDH | XBP-1  GAPDH | ATF6α  GAPDH |
| --- | --- | --- | --- | --- | --- | --- |
| Con | + | 0.14 | 0.23 | 0 | 0 | 0 |
| Sal | + | 0.32 | 0.35 | 0 | 0 | 0 |
| SB | + | 0.37 | 0.44 | 0.38 | 0.21 | 0.05 |
| SBSal | + | 0.65 | 0.54 | 0.21 | 0.14 | 0.04 |
| TM | + | 0.42 | 0.48 | 0.32 | 0.16 | 0.05 |
| TMSal | + | 0.60 | 0.66 | 0.19 | 0.13 | 0.05 |
| Con | - | 0.04 | 0.13 | 0 | 0 | 0 |
| Sal | - | 0.21 | 0.25 | 0 | 0 | 0 |
| SB | - | 0.26 | 0.24 | 0.24 | 0.09 | 0.02 |
| SBSal | - | 0.33 | 0.43 | 0.10 | 0.03 | 0.02 |
| TM | - | 0.29 | 0.38 | 0.13 | 0.05 | 0.02 |
| TMSal | - | 0.45 | 0.50 | 0.09 | 0.04 | 0.02 |
