# Supplementary material for: ER stress response plays an important role in aggregation of α-synuclein
Source: Mol Neurodegener. 2010 Dec 13;5:56. doi: 10.1186/1750-1326-5-56 (PMC3016345; doi:10.1186/1750-1326-5-56)
Supplement: Additional file 6 — Quantification of Western blot shown in additional file 5 [file 1750-1326-5-56-S6.DOC]

Additional file 6. Quantification of Western blot shown in Additional file 5.

|  | α-Syn | EIF2α  P/Total | GRP78  GAPDH | CHOP  GAPDH |
| --- | --- | --- | --- | --- |
| Con | + | 0.17 | 0.83 | 0 |
| ConSal | + | 0.25 | 1.08 | 0 |
| VPA | + | 0.86 | 0.85 | 0.52 |
| VPASal | + | 1.17 | 1.00 | 0.15 |
| Agk2 | + | 0.48 | 0.82 | 0.14 |
| Agk2Sal | + | 1.02 | 0.94 | 0.04 |
| Con | - | 0.03 | 0.35 | 0 |
| ConSal | - | 0.16 | 0.42 | 0 |
| VPA | - | 0.49 | 0.73 | 0.49 |
| VPASal | - | 0.73 | 0.80 | 0.20 |
| Agk2 | - | 0.15 | 0.58 | 0.12 |
| Agk2Sal | - | 0.44 | 0.82 | 0.05 |
