# Supplementary material for: ER stress response plays an important role in aggregation of α-synuclein
Source: Mol Neurodegener. 2010 Dec 13;5:56. doi: 10.1186/1750-1326-5-56 (PMC3016345; doi:10.1186/1750-1326-5-56)
Supplement: Additional file 8 — Quantification of Western blot shown in Figure 10 [file 1750-1326-5-56-S8.DOC]

Additional file 8. Quantification of Western blot shown in Figure 10.

|  | EIF2α  P/Total | GRP78  GAPDH | CHOP  GAPDH |
| --- | --- | --- | --- |
| TgCon | 0.50 | 0.80 | 0 |
| TgSal | 0.75 | 1.22 | 0 |
| TGSB | 0.73 | 2.71 | 0.70 |
| TGSBSal | 1.02 | 3.14 | 0.35 |
| NTCon | 0.44 | 0.05 | 0 |
| NTSal | 0.55 | 0.15 | 0 |
| NTSB | 0.64 | 1.53 | 0.41 |
| NTSBSal | 0.92 | 1.76 | 0.08 |
